# Treatment sequences and prognostic/predictive factors in metastatic pancreatic ductal adenocarcinoma: univariate and multivariate analyses of a real-world study in Europe

**DOI:** 10.1186/s12885-023-11377-1

**Published:** 2023-09-18

**Authors:** Julien Taieb, Thomas Seufferlein, Michele Reni, Daniel H. Palmer, John A. Bridgewater, Antonio Cubillo, Gerald W. Prager, Alice Vermeire, Fabienne Hédouin-Biville, Zhaoyang Teng, Teresa Macarulla

**Affiliations:** 1 Department of Gastroenterology and Digestive Oncology, Université de Paris, Georges Pompidou European Hospital, SIRIC CARPEM, Paris, France; 2 Université Paris-Cité, Hôpital Européen Georges Pompidou, Hepatogastroenterology and GI Oncology, Paris, France; 3 Department of Internal Medicine I, University Hospital Ulm, Ulm, Germany; 4 University Vita E Salute, IRCCS, San Raffaele Scientific Institute, Milan, Italy; 5 University of Liverpool, Liverpool, UK; 6 UCL Cancer Institute, London, UK; 7 HM CIOCC University Hospital, Madrid, Spain; 8 Department of Medicine I, Medical University of Vienna, Vienna, Austria; 9 Servier, Suresnes Cedex, France; 10 Servier Pharmaceuticals, Boston, USA; 11 Vall d´Hebron University Hospital, Vall d´Hebron Institute of Oncology (VHIO), IOB Quiron, Barcelona, Spain

**Keywords:** Pancreatic cancer, Metastatic, Survival, Prognostic factors, Real-world data

## Abstract

**Background:**

Real-world data on treatment patterns/outcomes for metastatic pancreatic cancer (mPAC) are limited. This study aims to assess real-world treatment patterns, survival outcomes, and prognostic/predictive factors in patients with mPAC.

**Methods:**

Retrospective, observational, chart-review involving medical oncologists and gastroenterologists from five European countries. Physicians reported information on disease and patient characteristics, diagnosis, and treatment for patients diagnosed with mPAC from January-October 2016. Outcomes included median progression-free survival (mPFS), median overall survival (mOS), and the impact of baseline performance status on survival. Univariate/multivariate regression analyses were undertaken to identify prognostic/predictive factors.

**Results:**

Three hundred four physicians and 3432 patients were included. First-line therapies included modified (m)FOLFIRINOX (28.4%), gemcitabine + nab-paclitaxel (28.0%), and gemcitabine monotherapy (23.0%). Frequent second-line therapies were gemcitabine monotherapy (25.0%), fluorouracil (5-FU) + oxaliplatin (21.8%), and gemcitabine + nab-paclitaxel (16.7%). Most frequent first- to second-line treatment sequences were gemcitabine + nab-paclitaxel followed by fluoropyrimidine combinations. Longest unadjusted estimated mOS was observed with (m)FOLFIRINOX followed by gemcitabine-based combinations (19.1 months). Multivariate analysis identified significant prognostic/predictive factors for OS and PFS including performance status and carbohydrate antigen 19–9 (CA 19–9) levels.

**Conclusions:**

Treatment and treatment sequences were generally in accordance with guidelines at the time of the study. Identification of prognostic/predictive factors for survival may help inform the individualised management of mPAC patients in the future.

**Supplementary Information:**

The online version contains supplementary material available at 10.1186/s12885-023-11377-1.

## Background

Metastatic pancreatic ductal adenocarcinoma (mPAC) is the fourth most frequent cause of cancer-related deaths in Europe [1], and by 2040, it is estimated that global incidence will increase by 77.7% and mortality will increase by 79.9% from 2018 levels [2]. mPAC is an aggressive disease and has one of the lowest survival prognoses [3], with an average 5-year survival rate of less than 10% [4, 5]. The aggressive nature of mPAC is due to a combination of factors, including a lack of early diagnostic markers, delayed detection due to lack of symptoms, complex genetic features and early metastatic spread [3, 6]. Surgical resection is the only potentially curative treatment for patients with mPAC [7, 8], and is recommended by the ESMO guidelines, with a 5-year survival rate of approximately 20% [3]. However, more than 80% of patients have an unresectable tumour at diagnosis [9], mainly due to vascular invasion and distant metastases, and therefore, sequential lines of chemotherapy are recommended by ESMO guidelines [3].

In the first-line treatment of mPAC, FOLFIRINOX (oxaliplatin, irinotecan, folinic acid and the fluoropyrimidine fluorouracil [5-FU]) has superior efficacy to gemcitabine alone in patients with a good Eastern Cooperative Oncology Group Performance Status (ECOG PS) of 0 or 1 – median overall survival (mOS) was significantly longer with FOLFIRINOX than with gemcitabine alone (11.1 months vs. 6.8 months; *p* < 0.001) [10]. It has also been demonstrated that the combination of gemcitabine plus nab-paclitaxel is superior to gemcitabine alone in patients with mPAC – mOS was 8.5 months with gemcitabine plus nab-paclitaxel and 6.7 months with gemcitabine monotherapy (*p* < 0.001) [11]. Thus, in patients with ECOG PS 0/1, ESMO 2015 guidelines recommended first-line treatment with either FOLFIRINOX or gemcitabine plus nab-paclitaxel [3]. Although not specifically recommended in these guidelines [3], patients with mPAC often receive modified (m)FOLFIRINOX in the first-line, which utilises a lower dose of chemotherapy than the standard regimen, and reduces toxicity [12]. More recently the publication of the PRODIGE 35 trial showed maintenance with folinic acid plus 5-FU appeared to be feasible and effective in patients with mPAC controlled after 4 months of induction chemotherapy with FOLFIRINOX [13]. Less fit patients (ECOG PS 2) are generally treated with first-line gemcitabine monotherapy or best supportive care, but some patients with ECOG PS 2 may receive gemcitabine plus nab-paclitaxel or reduced doses of FOLFIRINOX if the poor ECOG PS is due to a heavy tumour burden [3, 14].

In second-line treatment of mPAC, ESMO guidelines [3] highlighted that the efficacy of the combination of 5-FU, folinic acid and oxaliplatin showed mixed results following a gemcitabine-based first-line regimen [15, 16], and suggested the best evidence was for the use of liposomal irinotecan (when available) in combination with 5-FU and folinic acid in these patients [3, 17]. This was based on the data from the NAPOLI-1 trial published in 2016 (mOS was 6.1 months with liposomal irinotecan plus 5-FU and folinic acid and 4.2 months with 5-FU and folinic acid, *p* = 0.012) [18]. Liposomal irinotecan was subsequently approved by the EMA in October 2016 [19]. When FOLFIRINOX is used in the first-line, gemcitabine monotherapy or combination therapy is generally prescribed.

The optimal treatment sequence for patients with mPAC remains unclear, and few data are available regarding real-world treatment patterns and outcomes for mPAC in Europe. The objectives of this study were to identify real-world treatment patterns and sequences, assess survival outcomes according to treatment sequence, and to identify prognostic factors for survival in patients with mPAC. Data were collected for this analysis before the widespread availability of liposomal irinotecan, olaparib, pembrolizumab and NTRK gene fusion inhibitors for some patients with appropriate genetic alterations.

## Methods

### Study design

This retrospective, observational, chart review involved medical oncologists and gastroenterologists from France, Germany, Italy, Spain, and the UK. The chart review was conducted according to the Market Research Society Code of Conduct 2014. This code of conduct does not require any regulatory or ethics approvals [20]. Consent for physicians to participate in the study was obtained from their respective hospitals. All physicians provided their informed consent to participate in this study. Patient record forms (PRFs) were filled out by physicians without any direct contact with patients, and all information was anonymised. Data protection laws were adhered to (EU 2016/679 General Data Protection Regulation), and international codes of conduct were also followed.

### Study population

Physicians enrolled in this study were certified medical oncologists currently treating at least eight patients diagnosed with PAC over a 6-month period, and personally involved in making treatment decisions for these patients. Physicians were randomly invited to participate in the study and underwent a screening process (telephone or online) of eight questions, to confirm their practice and experience. Physicians were recruited from a range of regions and settings to ensure a balanced representation in each country (e.g., university and general hospitals, cancer and reference centres, and office-based specialists).

To avoid selection bias, the investigators were asked to record data for 20 consecutive patients diagnosed at their sites between 1^st^ January 2016 and 1^st^ October 2016. Patients were included if they were diagnosed with pancreatic adenocarcinoma and were 18 years of age or older at the time of diagnosis.

### Assessments

Physicians completed online PRFs electronically. Chart reports provided information on general disease and patient characteristics (age, sex, ECOG PS, location of primary tumour, histological data, and cancer antigen 19–9 levels [CA19-9]), diagnosis, and treatment type, including the entire patient history from diagnosis to 5 years or death, to generate data on diagnosis, treatment patterns, and survival outcomes. For this analysis, data were collected for all patients with mPAC, all patients who received first-line treatment (1L), all patients who received second-line treatment (2L), and all patients who received first-line followed by second-line treatment (1L → 2L). Information was collected for the treatments according to line of therapy and treatment sequences. (See Supplementary Table 1, Additional file 1). Follow-up data on progression and survival were collected as outlined below.

### Efficacy outcomes

Efficacy outcomes included median progression-free survival (PFS) and OS according to each line of therapy, baseline ECOG PS and treatment sequence. OS in the 1L and 1L → 2L populations was defined as the time from date of initiation of first-line treatment to date of death. OS in the 2L population was defined as the time from date of initiation of second-line treatment to date of death. For all OS analyses, in the absence of death confirmation, survival time was censored at the date the patient was last known alive. PFS in the 1L population was defined as the time from the date of initiation of first-line treatment until date of disease progression or death due to any cause. Patients who received second-line treatment before disease progression or death were censored at the end of the first-line treatment. Patients who were alive with no disease progression at first-line treatment and did not receive second-line treatment were censored at the end of the first-line treatment. If the end of the first-line treatment was missing, the patient’s last known alive date was used instead. PFS in the 2L population was defined as the time from date of initiation of second-line treatment until date of disease progression or death due to any cause. Patients who were alive with no disease progression at second-line treatment were censored at the end of second-line treatment. If the end of the second-line treatment was missing, the patient’s last known alive date was used instead.

### Statistics

Descriptive summary statistics (n, mean, median, minimum, and maximum) were provided for variables measured on a continuous scale. The frequency distribution (n, %) were provided for variables measured on a nominal scale. For each of the first- and second-line therapies, and sequences of treatment, Kaplan Meier survival analysis without covariates was utilised for PFS and OS. Unstratified Cox proportional hazards regression was used to estimate hazard ratios and their corresponding 95% confidence intervals. All *p*-values were based on a log-rank test to compare treatment groups.

Univariate analysis was performed using the Cox Proportional Hazard (CPH) model. Potential prognostic and predictive factors at baseline including first-line therapy used, second-line therapy used, age, gender, smoking status, alcohol consumption, body mass index [BMI], disease grading, number of comorbidities, CA19-9 status (≥ 400 or < 400 U/ml) [21, 22], location/spread of tumour, country, ECOG PS, presence of liver metastases, and presence of lung metastases were tested via the CPH for first- and second-line therapy. For the treatment sequence of 1L → 2L therapy, ECOG PS, presence of liver metastases, presence of lung metastases and CA19-9 were treated as timing-varying covariates, in which both the measures collected at the first-line therapy and the second-line therapy were used in univariate analysis. For CA19-9 status, if missing at the first-line therapy, the measure at metastatic setting was used instead. If the *p*-value was < 0.05, the prognostic/predictive factor was included in multivariate analysis (using the CPH model) to further identify a final subset of prognostic/predictive factors in the model. A sensitivity analysis was also conducted in order to explore the multivariate analysis of OS for second-line therapy due to the high number of missing data for CA19-9 levels at baseline. To test the assumption that Cox proportional hazards was met for variables in the multivariable analysis, in addition to interaction test, Schoenfeld residual plots were also generated for 1L and 2L treatments, which allows a visual assessment of the importance of potential violations of the proportional hazards assumption. In terms of the multivariate analysis for first- to -second-line therapy, the interaction test wasn’t conducted, since some time-varying covariates (PS, liver metastasis, lung metastasis and CA19-9 levels) were involved in the analysis. Instead, the Schoenfeld residue plots were provided for each covariate in the final multivariate model. SAS Version 9.4 was used for all analyses.

## Results

304 physicians (France [*n* = 62], Germany [*n* = 60], Italy [*n* = 63], Spain [*n* = 66], UK [*n* = 53]) participated in the collection of patient records. In total, 53.0% of centres across the five countries in Europe were academic/teaching/university hospitals, 26.3% were general hospitals, 9.5% were private hospitals or clinics, and the remaining 11.2% of sites were offices or ‘other’.

Of the 6000 PRFs collected, 3827 (63.8%) patients had mPAC at diagnosis, of which 3432 (89.7%) received a first-line treatment and 1218 (31.8%) received a second-line treatment (Table 1). Baseline characteristics were generally well balanced across the lines and sequences of treatment (Table 1) (See Supplementary Tables 2 and 3, Additional file 1).

**Table 1:** Characteristics of the population when starting first-line treatment and when starting second-line treatment

Characteristic	Population, n (%)^a^	
	**1L (*n* = 3432)**	**2L (*n* = 1218)**
Sex, Male	2041 (59.5)	732 (60.1)
Age, years, median (interquartile range)	65.3 (57.8, 72.4)	63.2 (56.7, 69.2)
Tumour location		
Head	1373 (40.0)	496 (40.7)
Body	785 (22.9)	279 (22.9)
Tail	297 (8.7)	114 (9.4)
Head/body	652 (19.0)	204 (16.7)
Body/tail	292 (8.5)	121 (9.9)
Unknown	33 (1.0)	4 (0.3)
CA19-9 level		
Median (interquartile range), U/ml	500 (148, 1805)	800 (300, 2800)
≥ 400 U/ml	1783 (52.0)	539 (44.2)
< 400 U/ml	1449 (42.2)	243 (20.0)
Missing	200 (5.8)	436 (35.8)
ECOG PS		
0	366 (10.7)	47 (3.9)
1	1830 (53.3)	568 (46.6)
2	1066 (31.1)	543 (44.6)
3	148 (4.3)	57 (4.7)
4	19 (0.6)	3 (0.2)
Unknown	3 (0.1)	0(0)

^a^Values are percentage unless otherwise stated in left-hand column

*CA19-9* Cancer antigen 19–9,  *ECOG PS* Eastern Cooperative Oncology Group Performance Status. *1L* First-line treatment, *2L* Second-line treatment

### Treatment use

**Fig. 1:**
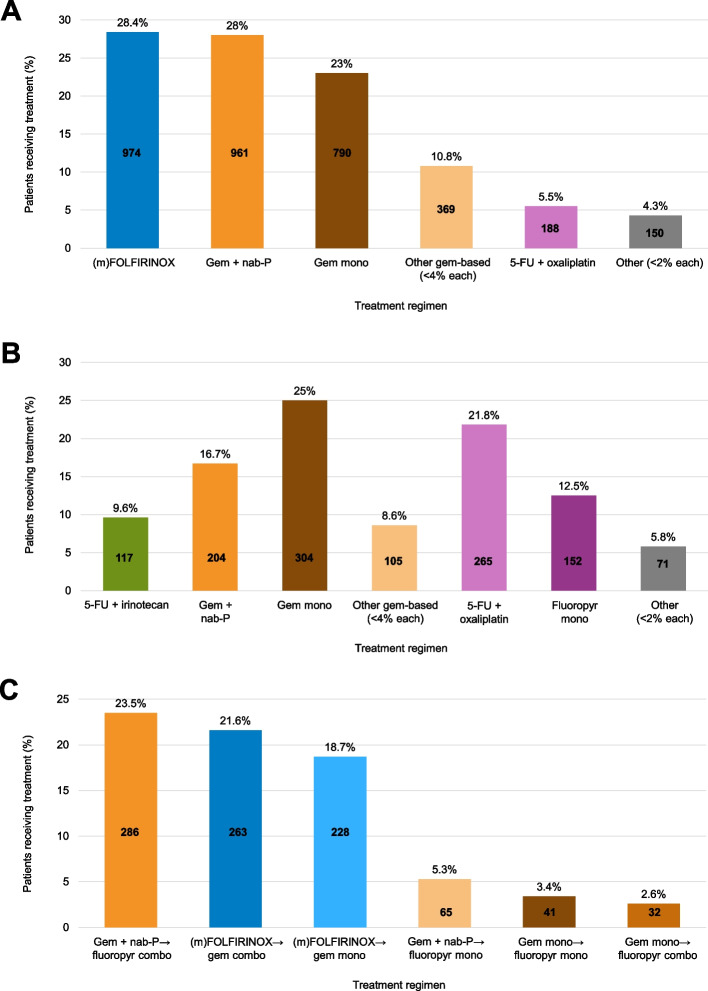
Most frequently prescribed treatment regimens in (**A**) first-line treatment, (**B**) second-line treatment, and (**C**) treatment sequences in first- and second-line. (m)FOLFIRINOX includes both standard and modified FOLFIRINOX. 5-FU, 5-fluorouracil. Combos, combinations. (m)FOLFIRINOX, modified folinic acid, fluorouracil, irinotecan and oxaliplatin. Fluoropyr, fluoropyrimidine. Gem, gemcitabine. Mono, monotherapy. nab-P, nab-paclitaxel

In the overall population, the most frequent first-line therapies were FOLFIRINOX (at the time of this chart review, centres were using standard and modified FOLFIRINOX – for simplicity, we subsequently refer to this as (m)FOLFIRINOX in the remainder of this manuscript), gemcitabine plus nab-paclitaxel, and gemcitabine monotherapy (Fig. 1A). The most frequent second-line therapies were gemcitabine monotherapy, 5-FU + oxaliplatin, and gemcitabine + nab-paclitaxel (Fig. 1B). The most frequent treatment sequences in first- to second-line were gemcitabine plus nab-paclitaxel followed by fluoropyrimidine combinations, (m)FOLFIRINOX followed by gemcitabine combinations or gemcitabine monotherapy (Fig. 1C). Of patients treated first with mFOLFIRINOX or gemcitabine plus nab-paclitaxel, 56% and 41% received a second-line therapy, respectively.

Second-line treatment choices were particularly variable between countries with the most frequent second-line therapies being gemcitabine plus nab-paclitaxel in Germany, 5-FU + oxaliplatin in Spain and Italy, gemcitabine monotherapy in France, and other gemcitabine combinations in the UK.

### Efficacy

**Fig. 2:**
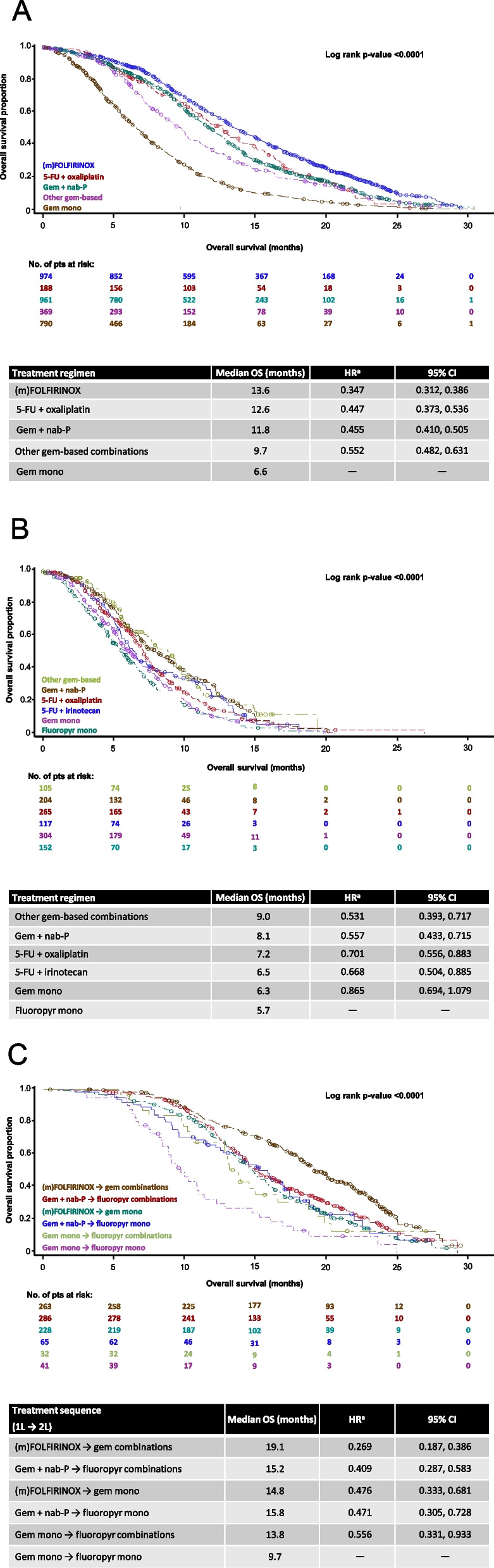
Kaplan–Meier overall survival curves (**A**) in the 1L population (*n* = 3432), (**B**) in the 2L population (*n* = 1218), and (**C**) in the 1L → 2L population (*n* = 1218). **A** (m)FOLFIRINOX includes both standard and modified FOLFIRINOX. ^a^HR with Gem mono as reference group. 5-FU, fluorouracil. CI, confidence interval. (m)FOLFIRINOX, modified folinic acid, fluorouracil, irinotecan and oxaliplatin. Gem, gemcitabine. Mono, monotherapy. HR, hazard ratio. nab-P, nab-paclitaxel. OS, overall survival. **B** ^a^HR with Fluoropyr mono as reference group. 5-FU, fluorouracil. Fluoropyr, fluoropyrimidine. CI, confidence interval. Gem, gemcitabine. HR, hazard ratio. Mono, monotherapy. nab-P, nab-paclitaxel. OS, overall survival. **C** (m)FOLFIRINOX includes both standard and modified FOLFIRINOX. ^a^HR with Gem mono followed by fluoropyr mono as reference group. (m)FOLFIRINOX, modified folinic acid, fluorouracil, irinotecan and oxaliplatin. CI, confidence interval. Fluoropyr, fluoropyrimidine. Gem, gemcitabine. HR, hazard ratio. Mono, monotherapy. nab-P, nab-paclitaxel. OS, overall survival

The longest mOS in the 1L population was observed with first-line (m)FOLFIRINOX (13.5 months; Fig. 2A). In the subgroup of patients with ECOG PS 0 or 1 at baseline, the longest mOS was observed with first-line (m)FOLFIRINOX (14.3 months; *n* = 839) and fluoropyrimidine + oxaliplatin (14.5 months; *n* = 141; See Supplementary Fig. 1A, Additional file 1). In the subgroup with ECOG PS ≥ 2 at baseline, the best mOS was achieved with first-line (m)FOLFIRINOX (10.0 months; *n* = 135; See Supplementary Fig. 1B, Additional file 1).

The longest mOS from the start of second-line treatment in the 2L population was observed with gemcitabine plus nab-paclitaxel (8.1 months; *n* = 204) and other gemcitabine-based combinations (9.0 months; *n* = 105; Fig. 2B). In those with ECOG PS 0 or 1 at baseline, second-line gemcitabine plus nab-paclitaxel (10.3 months) and other gemcitabine-based regimens (9.8 months) produced the longest mOS (See Supplementary Fig. 1C, Additional file 1). For patients with ECOG PS ≥ 2 at baseline, the longest mOS (6.3 months) was observed with second-line 5-FU plus oxaliplatin (See Supplementary Fig. 1D, Additional file 1).

In the 1L → 2L population, the longest unadjusted estimate of mOS was observed with the treatment sequence of (m)FOLFIRINOX followed by gemcitabine-based combination therapy (19.1 months; Fig. 2C). The same treatment sequence produced the longest mOS in patients with ECOG PS 0 or 1 at baseline (20.0 months; See Supplementary Fig. 1E, Additional file 1). In the subgroup with ECOG PS ≥ 2 at baseline, the longest mOS (15.0 months) was observed with the treatment sequence of (m)FOLFIRINOX followed by gemcitabine monotherapy (See Supplementary Fig. 1F, Additional file 1).

**Fig. 3:**
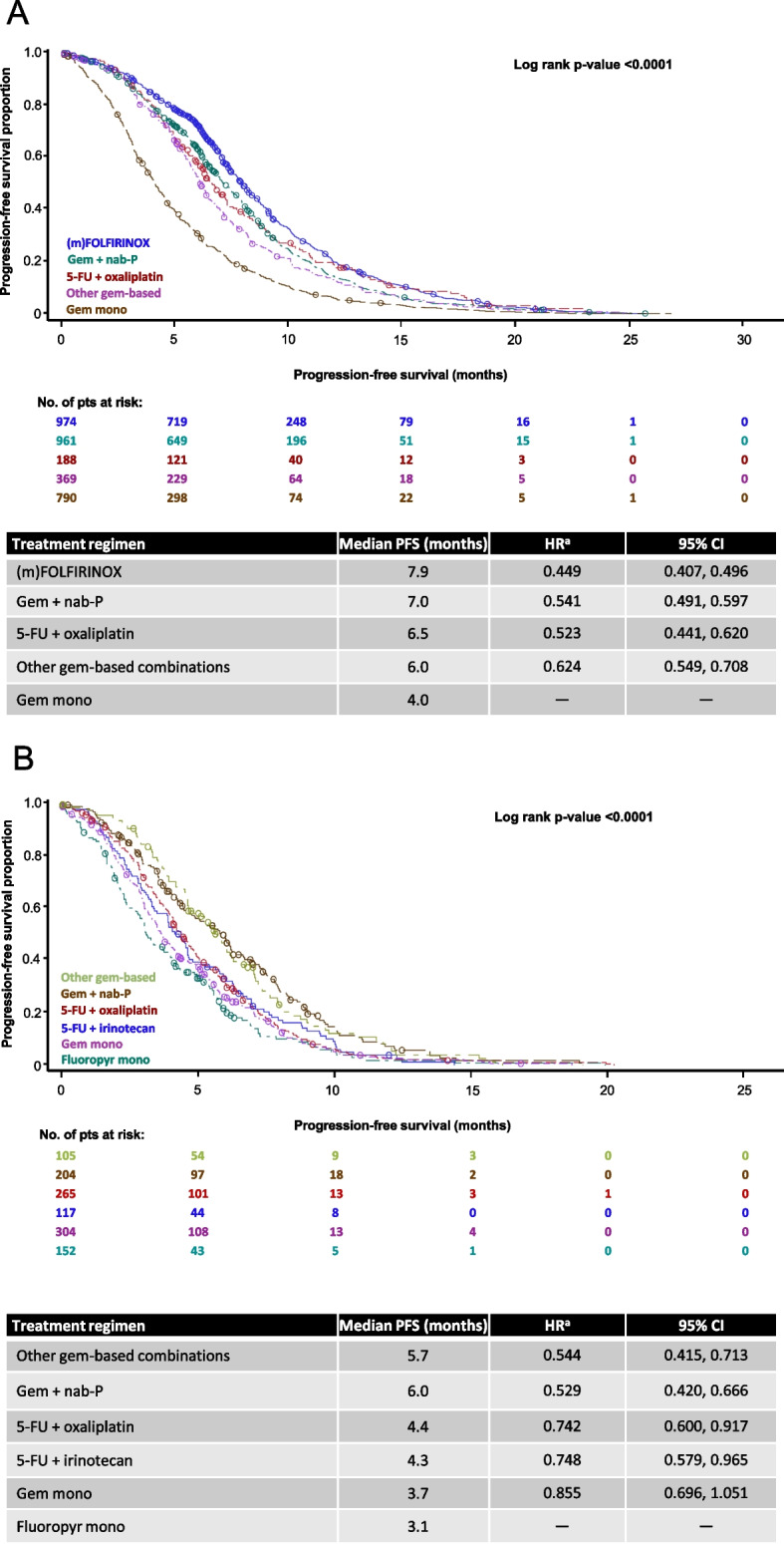
Kaplan–Meier curves for progression-free survival (A) in the 1L population (*n* = 3432), and (B) in the 2L population (*n* = 1218). **A** (m)FOLFIRINOX includes both standard and modified FOLFIRINOX. ^a^HR with Gem mono as reference group. 5-FU, fluorouracil. CI, confidence interval. (m)FOLFIRINOX, modified folinic acid, fluorouracil, irinotecan and oxaliplatin. HR, hazard ratio. Gem, gemcitabine. Mono, monotherapy. nab-P, nab-paclitaxel. PFS, progression-free survival. **B** (m)FOLFIRINOX includes both standard and modified FOLFIRINOX. ^a^HR with Fluoropyr mono as reference group. 5-FU, fluorouracil. CI, confidence interval. (m)FOLFIRINOX, modified folinic acid, fluorouracil, irinotecan and oxaliplatin. Fluoropyr, fluoropyrimidine. Gem, gemcitabine. HR, hazard ratio. Mono, monotherapy. nab-P, nab-paclitaxel. PFS, progression-free survival

In the 1L population, the longest mPFS was observed with first-line (m)FOLFIRINOX (7.82 months; Fig. 3A). The PFS findings in ECOG PS subgroups and in the 2L and 1L → 2L populations were broadly in-line with the mOS results (Fig. 3) (See Supplementary Fig. 2, Additional file 1).

### Prognostic/predictive factors

Univariate regression analysis was conducted to identify potential prognostic/predictive factors of OS for each population, and significant factors (with a *p* value of < 0.05) were used in the multivariate analyses (See Supplementary Table 4, Additional file 1). Multivariate analysis identified BMI, the presence of liver metastasis, ECOG PS, country of origin, age, disease grading, alcohol consumption, CA 19–9 levels and treatment regimen as independent prognostic/predictive factors for OS at first-line treatment (Table 2). At second-line, ECOG PS and CA 19–9 levels were identified as independent prognostic indicators for OS (Table 2). In the sensitivity analysis performed due to the high number of patients without data for CA 19–9 levels at second-line (782/1218 patients had data available at second-line), treatment, liver metastasis, CA19-9 at first-line, ECOG PS and tumour location were shown to be independent prognostic/predictive factors for OS at second-line (See Supplementary Table 5, Additional file 1).

**Table 2:** Prognostic/predictive factors for OS in multivariate analyses

Prognostic/predictive factors	HR (95% CI)	*p* value
**Multivariate analyses**		
**1L → 2L**		
Male vs. female	0.83 (0.70, 0.97)	0.0228
ECOG PS, 0/1 vs. ≥ 2	0.45 (0.38, 0.53)	< 0.0001
CA19-9, < 400 U/ml vs. ≥ 400 U/ml	0.75 (0.64, 0.88)	0.0004
Liver metastases, no vs. yes	0.40 (0.27, 0.59)	< 0.0001
Lung metastases, no vs. yes	0.79 (0.67, 0.93)	0.0049
**1L → 2L (vs. gem mono → Fluoropyr mono)**		
(m)FOLFIRINOX → Gem combinations	0.42 (0.29, 0.62)	< 0.0001
Gem + nab-P → Fluoropyr combinations	0.60 (0.42, 0.87)	
Gem + nab-P → Fluoropyr mono	0.65 (0.41, 1.01)	
(m)FOLFIRINOX → Gem mono	0.67 (0.46, 0.96)	
Gem mono → Fluoropyr combinations	0.79 (0.47, 1.33)	
**1L**		
Age, ≤ 70 vs. > 70 years	0.86 (0.78, 0.94)	0.0013
BMI, kg/m^2^		
18.5–25 vs. < 18.5	0.70 (0.61, 0.82)	< 0.0001
> 25 vs. < 18.5	0.67 (0.57, 0.80)	
Disease grading, 1 or 2 vs. 3 or 4	0.88 (0.81, 0.96)	0.0048
ECOG PS, 0/1 vs. ≥ 2	0.52 (0.48, 0.58)	< 0.0001
CA19-9, < 400 U/ml vs. ≥ 400 U/ml	0.92 (0.85, 0.99)	0.0369
Liver metastases, no vs. yes	0.70 (0.62, 0.80)	< 0.0001
Country		
Germany vs. UK	0.80 (0.70, 0.92)	< 0.0001
Spain vs. UK	0.85 (0.75, 0.96)	
France vs. UK	0.79 (0.70, 0.90)	
Italy vs. UK	0.73 (0.64, 0.84)	
Alcohol consumption		
Heavy vs. never/unknown	0.99 (0.84, 1.18)	0.0198
Moderate vs. never/unknown	0.88 (0.79, 0.99)	
Occasional vs. never/unknown	0.86 (0.77, 0.96)	
**1L (vs. gem mono)**		
(m)FOLFIRINOX	0.56 (0.49, 0.64)	< 0.0001
5-FU + oxaliplatin	0.70 (0.58, 0.85)	
Gem + nab-P	0.70 (0.62, 0.79)	
Gem other combinations	0.76 (0.66, 0.89)	
**2L**		
ECOG PS 0/1 vs. ≥ 2	0.52 (0.44, 0.61)	< 0.0001
CA19-9, < 400 U/ml vs. ≥ 400 U/ml	0.72 (0.60, 0.87)	0.0005

(m)FOLFIRINOX includes both standard and modified FOLFIRINOX

*5-FU* 5-fluorouracil, *BMI* Body mass index, *CA19-9* Cancer antigen 19–9, *CI* Confidence interval, *ECOG PS* Eastern Cooperative Oncology Group Performance Status, *(m)FOLFIRINOX* Modified folinic acid, fluorouracil, irinotecan and oxaliplatin, *Fluoropyr* Fluoropyrimidine, *Gem* Gemcitabine, *Mono* Monotherapy, *nab-P* Nab-paclitaxel, *OS* Overall survival, *1L* First-line treatment, *2L* Second-line treatment

In the 1L → 2L population, the presence of liver or lung metastasis, ECOG PS, CA19-9 levels, and male sex, were all significant and independent prognostic factors of OS (Table 2). Treatment sequence was also a significant predictive factor, with (m)FOLFIRINOX followed by gemcitabine combinations being the strongest favourable predictive factor (HR: 0.424; Table 2) with regard to treatment sequence.

Similar analyses of prognostic/predictive factors for PFS identified treatment regimen, ECOG PS, age, CA19-9 levels, BMI, liver metastasis and smoking status as independent prognostic/predictive factors at first-line treatment; and ECOG PS, treatment regimen, tumour location, disease grade and CA 19–9 levels at second-line treatment. Prognostic factors for second-line treatment were age, smoking status, alcohol consumption status, BMI, lung metastasis, liver metastasis, ECOG PS, country of origin and tumour location (Table 3).

**Table 3:** Logistic regression analysis of baseline characteristics associated with second-line administration

Variable	OR (95% CI)	*p* value
Age, ≤ 70 vs. > 70 years	1.50 (1.25, 1.80)	< 0.0001
Smoking status		
Current vs. never/unknown	1.34 (1.06, 1.70)	0.0440
Former vs. never/unknown	1.21 (0.99, 1.47)	
Alcohol consumption		
Heavy vs. never/unknown	0.62 (0.41, 0.93)	0.0037
Moderate vs. never/unknown	1.04 (0.81, 1.34)	
Occasional vs. never/unknown	1.20 (0.97, 1.49)	
BMI, kg/m^2^		
18.5–25 vs. < 18.5	2.27 (1.59, 3.23)	< 0.0001
> 25	2.93 (2.00, 4.31)	
Lung metastases, no vs. yes	1.49 (1.24, 1.78)	< 0.0001
ECOG PS, 0/1 vs. ≥ 2	5.63 (4.63, 6.85)	< 0.0001
Country		
Germany vs. UK	2.13 (1.64, 2.78)	< 0.0001
Spain vs. UK	2.33 (1.82, 2.98)	
France vs. UK	3.97 (3.06, 5.15)	
Italy vs. UK	1.37 (1.06, 1.77)	
Tumour location, body/tail or tail vs. head, body or head/body	1.27 (1.04, 1.55)	0.0221

*BMI* Body mass index, *CI* Confidence interval, *ECOG PS* Eastern Cooperative Oncology Group Performance Status, *HR* Hazard ratio

The interaction test between time and covariate was conducted for 1L treatment, and demonstrated that treatment type, gender and ECOG PS 1 were significant prognostic/predictive factors. However, Schoenfeld residual plots subsequently demonstrated that ECOG PS 1 was the only potential covariate that may have a minor violation of the proportional hazards assumption (see Supplementary Fig. 3, Additional file 1). For 2L treatment, the interaction test demonstrated that disease grade and ECOG PS 2 were significant prognostic/predictive factors, but Schoenfeld residual plots demonstrated that both covariates may have a potential minor violation of the proportional hazards assumption (see Supplementary Fig. 4, Additional file 1). For the first- to second-line treatment sequence, although violation of proportional hazards assumption is observed for CA19-9 and ECOG PS 1 (see Supplementary Fig. 5, Additional file 1), the proportional hazards assumption is not relevant as they have already been treated as timing-varying covariates in the multivariate analysis (see Supplementary Fig. 5, Additional file 1).

## Discussion

This large chart review exploring mPAC in the real-world setting demonstrated that the choice of first-line treatment among European physicians was generally in accordance with ESMO guidelines at the time of the study in 2016 [3]. (m)FOLFIRINOX and gemcitabine plus nab-paclitaxel were the most frequently prescribed first-line treatments, and most patients receiving these treatments had an ECOG PS of 0 or 1. Gemcitabine monotherapy was also frequently prescribed, but more often for patients with ECOG PS ≥ 2. Guidance on second-line treatment is less well defined in guidelines, and in the current analysis, the most frequent second-line therapies were gemcitabine monotherapy, 5-FU plus oxaliplatin, and gemcitabine + nab-paclitaxel. These findings are generally consistent with those of a real-world Dutch study, where patients were diagnosed with mPAC between 2015 and 2018: the most frequent 1L therapies were FOLFIRINOX, gemcitabine, and gemcitabine plus nab-paclitaxel; the most common 2L therapies were gemcitabine plus nab-paclitaxel, gemcitabine and FOLFIRINOX [23].

With different options for first-line treatment, the concept of optimising treatment sequences is emerging in mPAC, in the same way that it has emerged for colorectal cancer. It is important to understand that the choice of first-line treatment directly impacts the choice of second-line treatment, as in general, patients receive first-line 5-FU-based therapy followed by second-line gemcitabine-based therapy, or first-line gemcitabine-based therapy followed by second-line 5-FU-based therapy. Therefore, optimising the sequence of currently approved treatments is important, as it may lead to substantial OS improvements, urgently needed in this severe disease, and without the need for additional agents. In the current study, the most frequently prescribed treatment sequences in first- to -second-line were gemcitabine plus nab-paclitaxel followed by fluoropyrimidine combinations (23.5%); (m)FOLFIRINOX followed by gemcitabine combinations (21.6%); and (m)FOLFIRINOX followed by gemcitabine monotherapy (18.7%). Among the second-line fluoropyrimidine combinations used in patients treated with first-line gemcitabine plus nab-paclitaxel, FOLFOX was the most frequently prescribed (21.8%), followed by 5-FU monotherapy (12.5%; data not shown). In patients treated with first-line (m)FOLFIRINOX, gemcitabine monotherapy was the most frequent second-line treatment (25%), followed by gemcitabine + nab-paclitaxel (16.7%) and other gemcitabine-based combinations (8.6%; data not shown). In the aforementioned Dutch study, the most common treatment sequences in first- to second-line were: FOLFORINOX followed by gemcitabine with or without nab-paclitaxel; or gemcitabine followed by FOLFIRINOX or gemcitabine plus nab-paclitaxel [23], which is generally consistent with the findings of our own study.

The treatment pattern of first- and second-line treatment was also consistent with a recent real-world study on mPAC in nine European countries conducted between 2014 and 2016 [24]. This real-world study of treatment patterns in Europe demonstrated that first- and second-line therapy choices were in line with ESMO recommendations, but varied between different countries, due to influencing factors such as drug availability in first- and second-line, reimbursement and physician preference [24]. Such factors may have also influenced treatment choice in our study. The multivariate analysis in our study did suggest that the country had a significant impact on whether patients received second-line treatment and on mOS from first-line.

The longest mOS was observed with first-line (m)FOLFIRINOX, 5-FU plus oxaliplatin and gemcitabine plus nab-paclitaxel, consistent with the aforementioned Dutch study [23]. The proportion of patients with a good ECOG PS at baseline was higher among those receiving these therapies than those receiving other first-line chemotherapies, and this is likely to have had an impact on the mOS achieved. However, large phase III clinical trials also support benefits of FOLFIRINOX and gemcitabine plus nab-paclitaxel in terms of OS [10, 11], including the recent NAPOLI-3 trial results that support the use of triplet chemotherapy as first-line treatment [25], which is consistent with the study results shown here. Interestingly, in the subgroup of patients with ECOG PS ≥ 2 (as recorded in the medical records), first-line (m)FOLFIRINOX, as well as gemcitabine plus nab-paclitaxel, also resulted in longer mOS (10.0 and 9.0 months, respectively) than other first-line therapies, including gemcitabine monotherapy (5.9 months). This suggests that in this chart review, even patients with a poorer performance status can benefit from intensified treatment (with possible dose adaptations). This finding is in line with results from the FRAGRANCE trial demonstrating that gemcitabine plus nab-paclitaxel is a potential treatment option in metastatic pancreatic ductal adenocarcinoma patients with ECOG PS-2 [26]. However, other baseline factors such as bilirubin levels, and other outcomes such as adverse event frequency and dose modifications were not recorded in this review. Due to the retrospective nature of this series and the lack of many baseline data, these mOS findings in patients with ECOG PS ≥ 2 should be viewed with caution.

Several possible prognostic/predictive factors for OS and PFS in first- and second-line treatment, as well as for first- to second-line treatment sequences, were found in our analyses. However, Schoenfeld analysis demonstrated that ECOG PS (1L and 2L treatment) and disease grade (2L treatment) may have potential minor violations of the proportional hazards assumption for OS, and the strength of their prognostic/predictive value would need to be confirmed in future analyses. First- and second-line treatment options conferred a benefit according to the multivariate analyses, which confirmed the findings on mOS described above. CA 19–9 level, for example, has been shown to be a valuable prognostic factor that can be used to measure disease burden and potentially guide treatment decisions [27]. Consistent with this finding, in the current study, higher CA 19–9 levels (≥ 400 U/ml) were associated with a worse prognosis, and along with ECOG PS > 1, was the only poor prognostic factor for OS in both first- and second-line treatment.

There are limitations to this study: as this was a chart review and based on real-world data, there may be confounding factors that could have influenced outcomes, and other biases could potentially affect the results; changing regulatory environments may have influenced treatment choice over the study period; and the review was not designed to compare treatments, and thus, data on PFS and OS should be viewed accordingly. Furthermore, although it would have been of value to assess safety data for the various treatments and treatments sequences, the PRFs in this study did not record safety data, so this was not possible. However, this large cohort provides a clear picture of real-world treatment patterns in Europe five years ago, and the prognostic/predictive factors identified may help guide future treatment decisions. Further research into the validity of these prognostic/predictive factors in mPAC will provide invaluable support in personalising treatment combinations and sequences. Since 2016, new treatment options have become available for some patients (e.g., liposomal-irinotecan, olaparib for germline BRCA mPAC, pembrolizumab for microsatellite instability high tumours, other PARP inhibitors or NTRK gene fusion inhibitors for NTRK-fusion positive mPAC) and have been added to international guidelines. For example, a real-world study conducted in Austria where patients with mPAC were treated between 2016 and 2018 demonstrated the use of liposomal-irinotecan in combination with 5-FU and folinic acid following gemcitabine-based therapies [28]. The impact of olaparib on survival in the real-world setting in mPAC has also been reported [29]. However, in general, treatment patterns have not changed dramatically since the current study was conducted [30]. Therefore, there is the potential that survival improvements have been achieved with sequential therapy in recent years. Continued awareness of current ESMO guidelines, and prognostic/predictive factors for survival, may support improved patient management decisions in the future.

## Conclusion

In conclusion, choice of first- and second-line treatments for patients with mPAC and treatment sequences from first- to second-line were generally in accordance with guidelines at the time of the study (2016). Longest mOS was observed with (m)FOLFIRINOX followed by gemcitabine-based combinations, indicating that intensive treatment may be beneficial. Treatment choice and sequence was a good predictor of mOS in this study, and ECOG PS and CA 19–9 were consistent predictors of OS in first- and second-line settings. Identification of prognostic/predictive factors for survival may help inform the individualised management of mPAC patients in the future.

### Supplementary Information

**Additional file 1:** **Supplementary Table 1.** Groupings for first and second-line treatments, and treatment sequences collected in the current analysis. **Supplementary Table 2.** Baseline characteristics of patients who received first-line followed by second-line treatment (1L→2L). **Supplementary Table 3.** Baseline characteristics in individual first-line treatment groups. **Supplementary Table 4.** Prognostic/predictive factors for overall survival in univariate analyses. **Supplementary Table 5.** Prognostic/predictive factors for OS at second-line in sensitivity analysis. **Supplementary Figure 1.** Kaplan-Meier overall survival curves (A) in the 1L population with ECOG PS 0 or 1 (*n*=2127), (B) in the 1L population with ECOG PS ≥2 (*n*=1153), (C) in the 2L population with ECOG PS 0 or 1 (*n*=563), (D) in the 2L population with ECOG PS ≥2 (*n*=584), (E) in the 1L→2L population with ECOG 0 or 1, and (F) in the 1L→2L population with ECOG PS ≥2. **Supplementary Figure 2.** Kaplan-Meier curves for progression-free survival (A) in the 1L population with ECOG PS 0 or 1 (*n*=2127), (B) in the 1L population with ECOG PS ≥2 (n=1153), (C) in the 2L population with ECOG PS 0 or 1 (*n*=563), and (D) in the 2L population with ECOG PS ≥2 (*n*=584).**Supplementary Figure 3.** Schoenfeld residual plots for potential variables not meeting the proportional hazard assumption from the interaction test: 1L therapy. (A) 1L (m)FOLFIRINOX; (B) 1L 5-FU + oxaliplatin; (C) 1L gemcitabine + nab-paclitaxel; (D) 1L gemcitabine monotherapy; (E) female gender; (F) ECOG PS 1. **Supplementary Figure 4.** Schoenfeld residual plots for potential variables not meeting the proportional hazard assumption from the interaction test: 2L therapy. (A) disease grade; (B) ECOG PS 2. **Supplementary Figure 5.** Schoenfeld residual plots for covariables: 1L→2L therapy. (A) 1L (m)FOLFIRINOX→2L gemcitabine monotherapy; (B) 1L (m)FOLFIRINOX→2L gemcitabine-based combinations; (C) 1L gemcitabine + nab-paclitaxel→2L fluoropyrimidine monotherapy; (D) 1L gemcitabine + nab-paclitaxel→2L fluoropyrimidine-based combinations; (E) 1L gemcitabine monotherapy→2L fluoropyrimidine monotherapy; (F) age; (G) female gender; (H) BMI <18.5 kg/m^2^; (I) BMI 18.5-25 kg/m^2^; (J) lung metastasis; (K) liver metastasis; (L) disease grade; (M) morbidity; (N) CA19-9≥400 U/ml; (O) ECOG PS 1; (P) tumour location.

## Data Availability

Data are not publicly available in a database form; however, data can be made available upon reasonable request from a qualified medical or scientific professional, for the specific purpose laid out in that request, and may include de-identified individual participant data. The data for this request will be available after a data access agreement has been signed. Any data-sharing requests should be sent to the following Data Request Portal: https://clinicaltrials.servier.com/data-request-portal. Access to patient-level data depends on a number of constraints, such as the year the study was performed, and an anonymization procedure. Requests are reviewed by a qualified panel of Servier experts and, if necessary, by an independent review board, and decisions will be communicated within three months, as detailed on the Data Request Portal mentioned above.

## Abbreviations

BMI: Body mass index
CA 19-9: Carbohydrate antigen 19–9
CPH: Cox Proportional Hazard
ECOG PS: Eastern Cooperative Oncology Group Performance Status
mOS: Median overall survival
mPAC: Metastatic pancreatic cancer
mPFS: Median progression-free survival
1L: First-line
1L → 2L: First-line followed by second-line treatment
2L: Second-line
5-FU: Fluorouracil
