# Supplementary material for: Treatment sequences and prognostic/predictive factors in metastatic pancreatic ductal adenocarcinoma: univariate and multivariate analyses of a real-world study in Europe
Source: BMC Cancer. 2023 Sep 18;23:877. doi: 10.1186/s12885-023-11377-1 (PMC10506331; doi:10.1186/s12885-023-11377-1)
Supplement: Supplementary file 1 — Additional file 1: Supplementary Table 1. Groupings for first and second-line treatments, and treatment sequences collected in the current analysis. Supplementary Table 2. Baseline characteristics of patients who received first-line followed by second-line treatment (1L→2L). Supplementary Table 3. Baseline characteristics in individual first-line treatment groups. Supplementary Table 4. Prognostic/predictive factors for overall survival in univariate analyses. Supplementary Table 5. Prognostic/predictive factors for OS at second-line in sensitivity analysis. Supplementary Figure 1. Kaplan-Meier overall survival curves (A) in the 1L population with ECOG PS 0 or 1 (n=2127), (B) in the 1L population with ECOG PS ≥2 (n=1153), (C) in the 2L population with ECOG PS 0 or 1 (n=563), (D) in the 2L population with ECOG PS ≥2 (n=584), (E) in the 1L→2L population with ECOG 0 or 1, and (F) in the 1L→2L population with ECOG PS ≥2. Supplementary Figure 2. Kaplan-Meier curves for progression-free survival (A) in the 1L population with ECOG PS 0 or 1 (n=2127), (B) in the 1L population with ECOG PS ≥2 (n=1153), (C) in the 2L population with ECOG PS 0 or 1 (n=563), and (D) in the 2L population with ECOG PS ≥2 (n=584).Supplementary Figure 3. Schoenfeld residual plots for potential variables not meeting the proportional hazard assumption from the interaction test: 1L therapy. (A) 1L (m)FOLFIRINOX; (B) 1L 5-FU + oxaliplatin; (C) 1L gemcitabine + nab-paclitaxel; (D) 1L gemcitabine monotherapy; (E) female gender; (F) ECOG PS 1. Supplementary Figure 4. Schoenfeld residual plots for potential variables not meeting the proportional hazard assumption from the interaction test: 2L therapy. (A) disease grade; (B) ECOG PS 2. Supplementary Figure 5. Schoenfeld residual plots for covariables: 1L→2L therapy. (A) 1L (m)FOLFIRINOX→2L gemcitabine monotherapy; (B) 1L (m)FOLFIRINOX→2L gemcitabine-based combinations; (C) 1L gemcitabine + nab-paclitaxel→2L fluoropyrimidine monotherapy; (D) 1L gemcitabine + [file 12885_2023_11377_MOESM1_ESM.docx]

**Additional File 1 to:**

**Treatment sequences and prognostic/predictive factors in metastatic pancreatic ductal adenocarcinoma: univariate and multivariate analyses of a real-world study in Europe**

**Supplementary Table 1.** Groupings for first and second-line treatments, and treatment sequences collected in the current analysis

| **First-line** | **Second-line** | **First-line → second-line** |
| --- | --- | --- |
| (m)FOLFIRINOX | 5-FU + irinotecan | (m)FOLFIRINOX → gem combinations |
| 5-FU + oxaliplatin | 5-FU + oxaliplatin | (m)FOLFIRINOX → gem mono |
| Gem + nab-P | Fluoropyr mono | Gem + nab-P → fluoropyr combinations |
| Gem mono | Gem + nab-P | Gem + nab-P → fluoropyr mono |
| Other gem-based combinations | Gem mono | Gem mono → fluoropyr combinations |
| Other | Other gem-based combinations | Gem mono → fluoropyr mono |

(m)FOLFIRINOX includes both standard and modified FOLFIRINOX. 5-FU, fluorouracil. (m)FOLFIRINOX, modified folinic acid, fluorouracil, irinotecan and oxaliplatin. Fluoropyr, fluoropyrimidine. Gem, gemcitabine. Mono, monotherapy. nab-P, nab-paclitaxel.

**Supplementary Table 2.** Baseline characteristics of patients who received first-line followed by second-line treatment (1L→2L)

| **Characteristic** | **1L→2L**  **N (%)**  **N=1218** | |
| --- | --- | --- |
| Sex, Male | 732 (60.1) | |
| Age, median, years | 63.2 | |
| Tumour location | | |
| Head | 496 (40.7) | |
| Body | 279 (22.9) | |
| Tail | 114 (9.4) | |
| Head/body | 204 (16.7) | |
| Body/tail | 121 (9.9) | |
| Unknown | 4 (0.3) | |
|  | **Start of 1L** | **Start of 2L** |
| CA19-9 level | | |
| ≥400 U/ml | 627 (51.5) | 539 (44.2) |
| <400 U/ml | 536 (44.0) | 243 (20.0) |
| Missing | 55 (4.5) | 436 (35.8) |
| ECOG PS | | |
| 0 | 204 (16.7) | 47 (3.9) |
| 1 | 830 (68.1) | 568 (46.6) |
| 2 | 170 (14.0) | 543 (44.6) |
| 3 | 13 (1.1) | 57 (4.7) |
| 4 | 1 (0.1) | 3 (0.2) |
| Unknown | 0 (0) | 0 (0) |

^a^Values are percentage unless otherwise stated in left-hand column.
CA19-9, cancer antigen 19-9. ECOG PS, Eastern Cooperative Oncology Group Performance Status. 1L, first-line treatment. 2L, second-line treatment.

**Supplementary Table 3.** Baseline characteristics in individual first-line treatment groups

| **Characteristic** | **1L treatment, n (%)^a^ N=3432** | | | | |
| --- | --- | --- | --- | --- | --- |
|  | **(m)FOLFIRINOX** | **5-FU + ox** | **Gem + nab-P** | **Gem + other** | **Gem mono** |
| Gender  Male Female | 635 (65.2) 330 (34.8) | 116 (61.7) 72 (38.3) | 567 (59.0) 394 (41.0) | 208 (56.4) 161 (43.6) | 428 (54.2) 362 (45.8) |
| ECOG PS  0/1 ≥2 | 839 (86.1) 135 (13.9) | 141 (75^b^) 45 (23.9^b^) | 704 (73.2) 257 (26.7) | 224 (60.7) 145 (39.3) | 219 (27.7) 571 (72.3) |
| Age at mPAC, years | 60.7 | 62.6 | 65.4 | 65.2 | 73.3 |
| CA19-9 status  <400 U/ml ≥400 U/ml | 68 (44.2) 86 (55.8) | 11 (36.7) 19 (63.3) | 68 (38.0) 111 (62.0) | 39 (67.2) 19 (32.8) | 38 (33.6) 75 (66.4) |

(m)FOLFIRINOX includes both standard and modified FOLFIRINOX. ^a^Values are percentage unless otherwise stated in left-hand column. ^b^ECOG PS data was missing for some patients (1.1%) in the fluoropyr + oxaliplatin groups. 5-FU, 5-fluorouracil. CA19-9, cancer antigen 19-9. ECOG-PS, ECOG PS, Eastern Cooperative Oncology Group Performance Status. Gem, gemcitabine. Mono, monotherapy. (m)FOLFIRINOX, modified folinic acid, fluorouracil, irinotecan and oxaliplatin. mPAC, metastatic pancreatic cancer. nab-P, nab-paclitaxel. 1L, first-line treatment.

**Supplementary Table 4.** Prognostic/predictive factors for overall survival in univariate analyses

| **Prognostic factors** | **p value** |
| --- | --- |
| **1L population** | |
| First-line treatment regimen | <0.0001 |
| Age (≤70 vs. >70 years) | <0.0001 |
| Sex | 0.0166 |
| Smoking (current, former, or never/unknown) | 0.0403 |
| Alcohol consumption (moderate, occasional or never/unknown vs. heavy) | 0.0002 |
| Body mass index (>25 or ≥18.5–25 vs. <18.5) | <0.0001 |
| Disease grade (1 or 2 vs. 3 or 4) | <0.0001 |
| Liver metastases (no vs. yes) | <0.0001 |
| Lung metastases (no vs. yes) | 0.0054 |
| Comorbidity (≤mean vs. >mean) | <0.0001 |
| Tumour location (body/tail or tail vs. head, body or head/body) | 0.5429 |
| ECOG PS (0–1 vs. ≥2) | <0.0001 |
| CA19-9 level (<400 U/ml vs. ≥400 U/ml) | 0.0002 |
| Country (Germany, France, Italy, Spain, UK) | 0.0002 |
| **2L population** | |
| Second-line treatment regimen | <0.0001 |
| Age (≤70 vs. >70 years) | 0.0449 |
| Sex | 0.0565 |
| Smoking (current, former, or never/unknown) | 0.5503 |
| Alcohol consumption (moderate, occasional or never/unknown vs. heavy) | 0.5052 |
| Body mass index (>25 or ≥18.5-25 vs. <18.5) | 0.0014 |
| Disease grade (1 or 2 vs. 3 or 4) | 0.0131 |
| Liver metastases (no vs. yes) | <0.0001 |
| Lung metastases (no vs. yes) | 0.0194 |
| Comorbidity (≤mean vs. >mean) | 0.0046 |
| Tumour location (body/tail or tail vs. head, body or head/body) | 0.0076 |
| ECOG PS (0-1 vs. ≥2) | <0.0001 |
| CA19-9 level (<400 U/ml vs. ≥400 U/ml) | 0.0003 |
| Country (Germany, France, Italy, Spain, UK) | 0.0840 |
| **1L→2L population** | |
| First-line treatment regimen and second-line regimen | <0.0001 |
| Age (≤70 vs. >70 years) | <0.0001 |
| Sex | 0.0049 |
| Smoking (current, former, or never/unknown) | 0.1000 |
| Alcohol consumption (moderate, occasional or never/unknown vs. heavy) | 0.0169 |
| Body mass index (>25 or ≥18.5–25 vs. <18.5) | 0.0004 |
| Disease grade (1 or 2 vs. 3 or 4) | 0.0005 |
| Liver metastases (no vs. yes) | <0.0001 |
| Lung metastases (no vs. yes) | <0.0001 |
| Comorbidity (≤mean vs. >mean) | <0.0001 |
| Tumour location (body/tail or tail vs. head, body or head/body) | 0.0439 |
| ECOG PS (0­–1 vs. ≥2) | <0.0001 |
| CA19-9 level (<400 U/ml vs. ≥400 U/ml) | <0.0001 |
| Country (Germany, France, Italy, Spain, UK) | 0.1858 |

CA19-9, cancer antigen 19-9. ECOG-PS, ECOG PS, Eastern Cooperative Oncology Group Performance Status. 1L, first-line treatment. 2L, second-line treatment.

**Supplementary Table 5.** Prognostic/predictive factors for OS at second-line in sensitivity analysis

| **Prognostic/predictive factors** | **HR (95% CI)** | **p value** |
| --- | --- | --- |
| 2L treatment, vs Fluoropyr mono | | |
| 5-FU + oxaliplatin | 0.797 (0.628, 1.011) | 0.0003 |
| 5-FU + irinotecan | 0.742 (0.554, 0.994) |  |
| Gem + nab-P | 0.565 (0.437, 0.731) |  |
| Other gem-based combinations | 0.656 (0.479, 0.899) |  |
| Gem mono | 0.854 (0.680, 1.073) |  |
| CA19-9 level (<400 U/ml vs. ≥400 U/ml) | 0.764 (0.662, 0.882) | 0.0002 |
| Liver metastases, no vs. yes | 0.594 (0.426, 0.827) | 0.0021 |
| ECOG PS, 0/1 vs. ≥2 | 0.577 (0.499, 0.668) | <0.0001 |
| Tumour location: head, body or head/body vs. body/tail or tail | 0.840 (0.706, 0.999) | 0.0481 |

5-FU, 5-flurouracil. CA19-9, cancer antigen 19-9. ECOG-PS, ECOG PS, Eastern Cooperative Oncology Group Performance Status. Fluoropyr, fluoropyrimidine. Gem, gemcitabine. Mono, monotherapy. nab-P, nab-paclitaxel. CI, confidence interval; HR, hazard ratio.

**Supplementary Figure 1.** Kaplan-Meier overall survival curves (A) in the 1L population with ECOG PS 0 or 1 (n=2127), (B) in the 1L population with ECOG PS ≥2 (n=1153), (C) in the 2L population with ECOG PS 0 or 1 (n=563), (D) in the 2L population with ECOG PS ≥2 (n=584), (E) in the 1L→2L population with ECOG 0 or 1, and (F) in the 1L→2L population with ECOG PS ≥2.

A


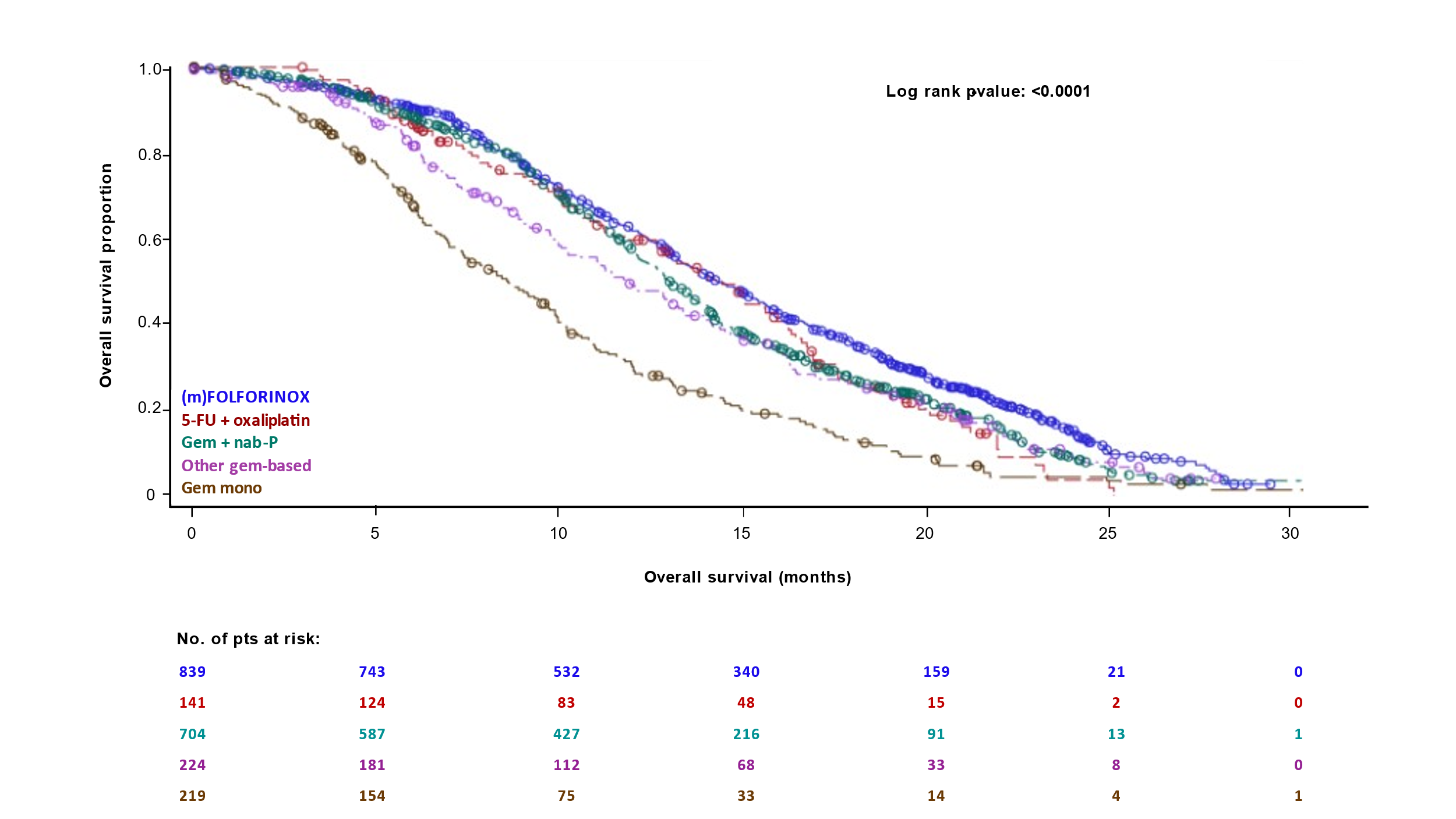


| **Treatment regimen** | **Median OS (months)** | **95% CI** |
| --- | --- | --- |
| (m)FOLFIRINOX | 14.3 | 13.57, 15.21 |
| 5-FU + oxaliplatin | 14.5 | 12.65, 15.93 |
| Gem + nab-P | 13.0 | 12.45, 13.67 |
| Other gem-based combinations | 11.8 | 10.25, 13.37 |
| Gem mono | 8.6 | 7.33, 9.89 |

(m)FOLFIRINOX includes both standard and modified FOLFIRINOX. 5-FU, fluorouracil. CI, confidence interval. (m)FOLFIRINOX, modified folinic acid, fluorouracil, irinotecan and oxaliplatin. Gem, gemcitabine. Mono, monotherapy. nab-P, nab-paclitaxel. OS, overall survival.

B


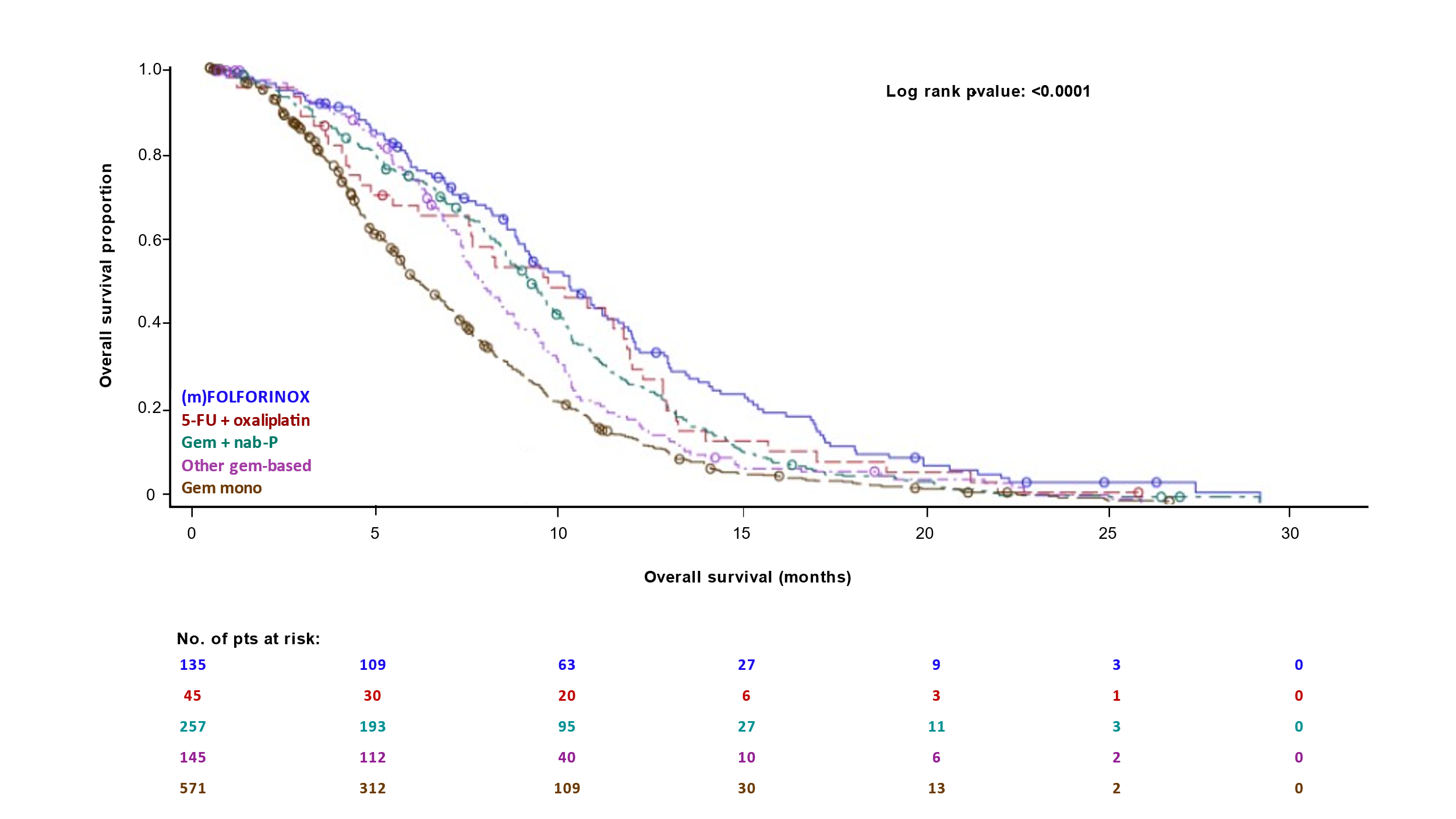


| **Treatment regimen** | **Median OS (months)** | **95% CI** |
| --- | --- | --- |
| (m)FOLFIRINOX | 10.0 | 8.77, 11.37 |
| 5-FU + oxaliplatin | 9.4 | 7.23, 11.63 |
| Gem + nab-P | 9.1 | 8.28, 9.59 |
| Other gem-based combinations | 7.6 | 7.06, 8.51 |
| Gem mono | 5.9 | 5.42, 6.57 |

(m)FOLFIRINOX includes both standard and modified FOLFIRINOX. (m)FOLFIRINOX, modified folinic acid, fluorouracil, irinotecan and oxaliplatin. 5-FU, 5-fluorouracil. CI, confidence interval. Gem, gemcitabine. Mono, monotherapy. nab-P, nab-paclitaxel. OS, overall survival.

C


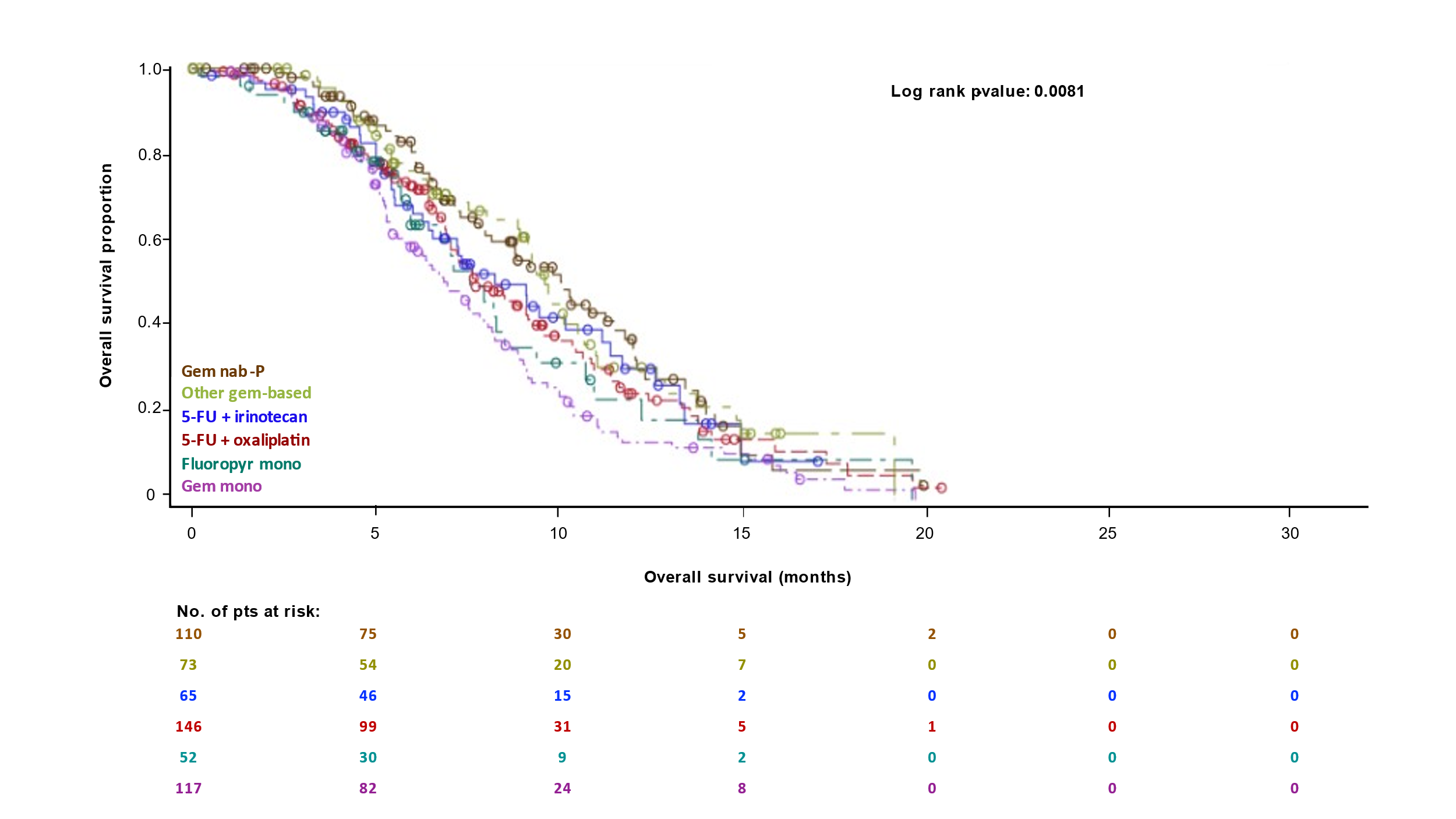


| **Treatment regimen** | **Median OS (months)** | **95% CI** |
| --- | --- | --- |
| Gem + nab-P | 10.3 | 8.11, 11.96 |
| Other gem-based combinations | 9.8 | 9.00, 10.84 |
| 5-FU + irinotecan | 8.4 | 6.54, 11.56 |
| 5-FU + oxaliplatin | 7.9 | 7.16, 9.43 |
| Fluoropyr mono | 7.7 | 5.98, 9.53 |
| Gem mono | 7.1 | 5.88, 8.18 |

(m)FOLFIRINOX includes both standard and modified FOLFIRINOX. 5-FU, 5-fluorouracil. CI, confidence interval. (m)FOLFIRINOX, modified folinic acid, fluorouracil, irinotecan and oxaliplatin. Fluoropyr, fluoropyrimidine. Gem, gemcitabine. Mono, monotherapy. nab-P, nab-paclitaxel. OS, overall survival.

D


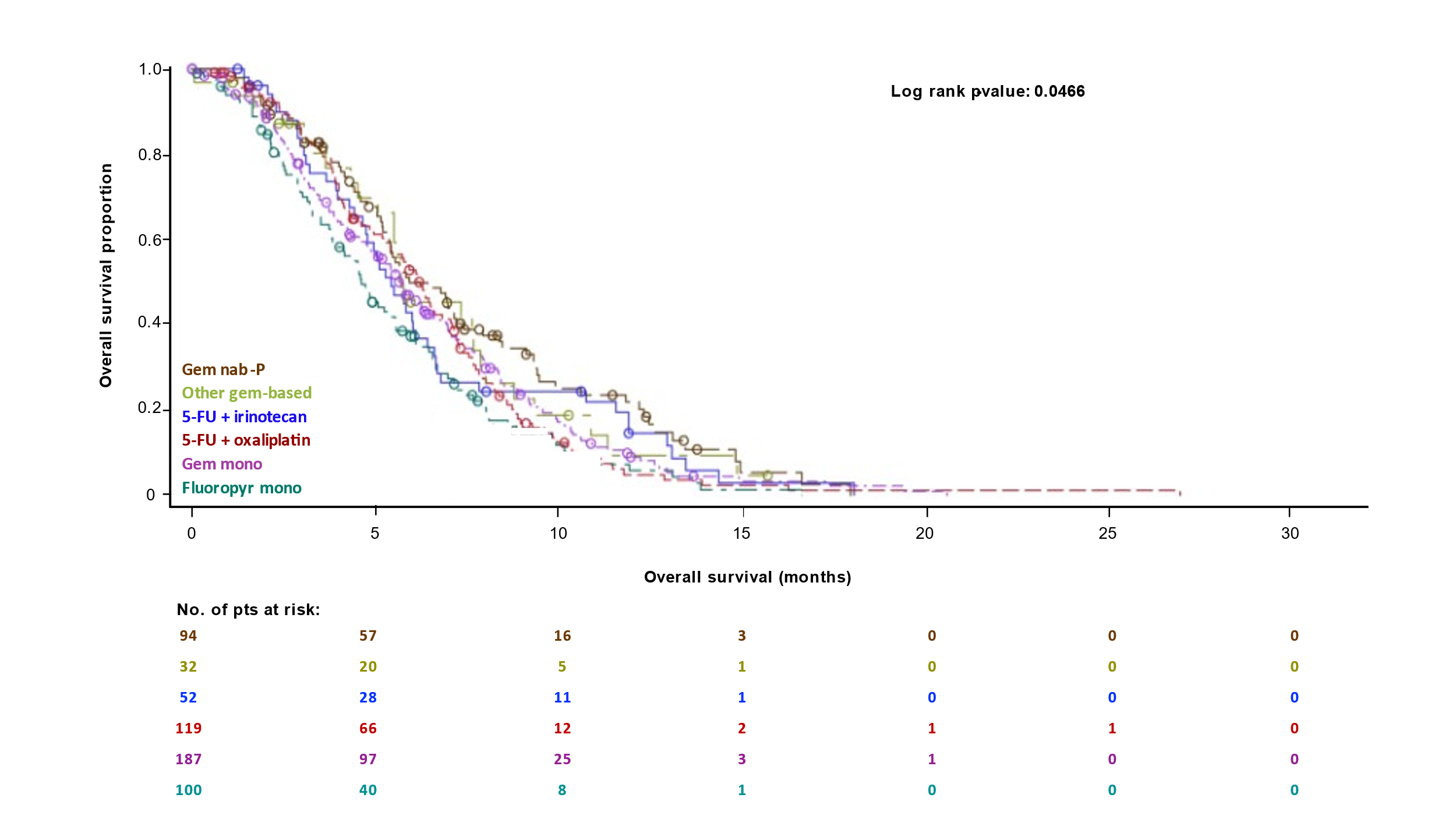


| **Treatment regimen** | **Median OS (months)** | **95% CI** |
| --- | --- | --- |
| Gem + nab-P | 6.0 | 5.39, 7.46 |
| Other gem-based combinations | 5.7 | 4.57, 7.92 |
| 5-FU + irinotecan | 5.5 | 4.70, 6.47 |
| 5-FU + oxaliplatin | 6.3 | 5.32, 7.10 |
| Gem mono | 5.8 | 4.93, 6.41 |
| Fluoropyr mono | 4.7 | 3.81, 5.59 |

(m)FOLFIRINOX includes both standard and modified FOLFIRINOX. 5-FU, fluorouracil. CI, confidence interval. (m)FOLFIRINOX, modified folinic acid, fluorouracil, irinotecan and oxaliplatin. Fluoropyr, fluoropyrimidine. Gem, gemcitabine. Mono, monotherapy. nab-P, nab-paclitaxel. OS, overall survival.

E


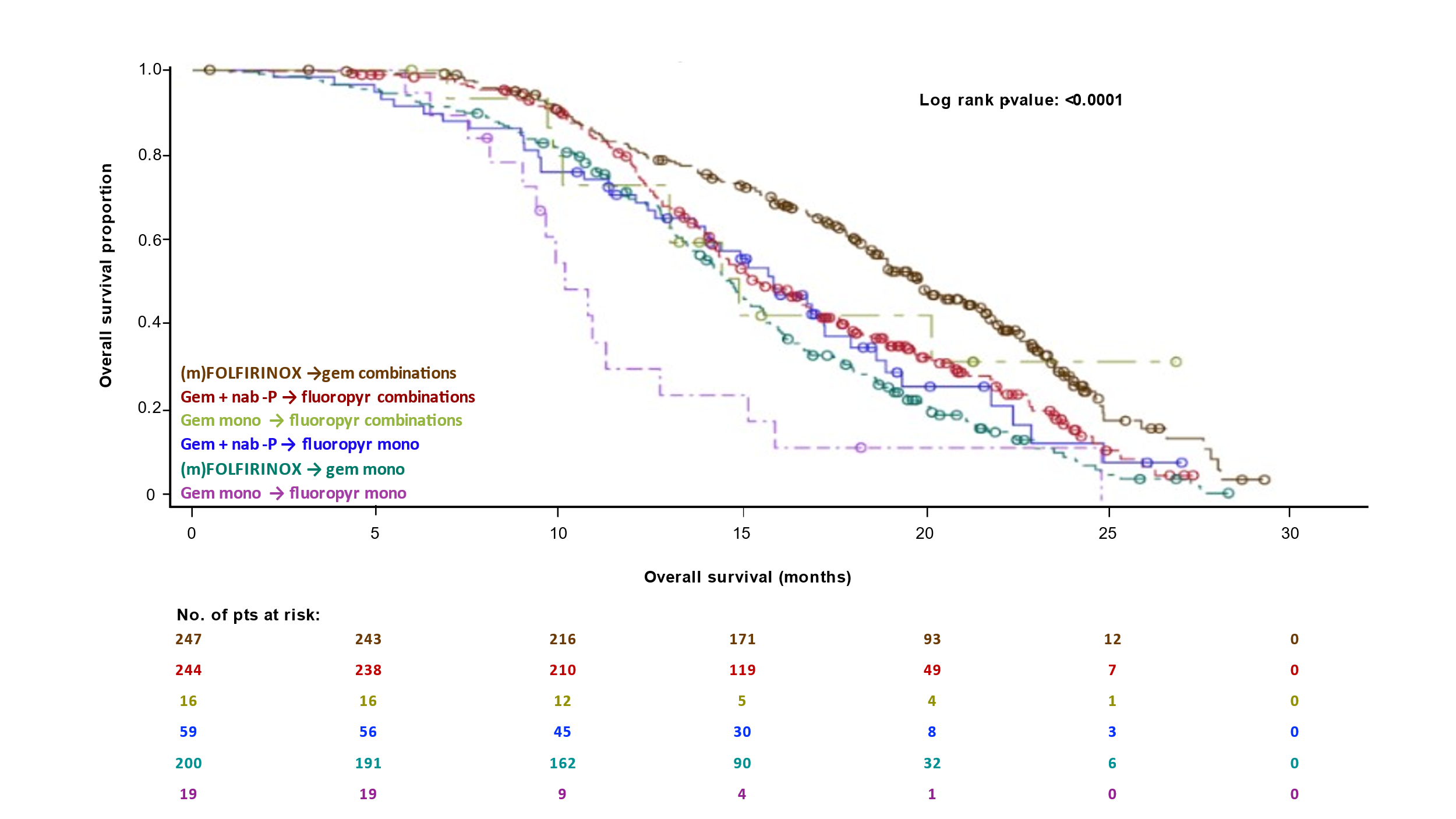


| **Treatment sequence (1L → 2L)** | **Median OS (months)** | **95% CI** |
| --- | --- | --- |
| (m)FOLFIRINOX → gem combinations | 20.0 | 18.83, 21.82 |
| Gem + nab-P → Fluoropyr combinations | 15.6 | 14.42, 16.95 |
| Gem mono → Fluoropyr combinations | 15.0 | 9.89, — |
| Gem + nab-P → Fluoropyr mono | 15.9 | 13.77, 18.04 |
| (m)FOLFIRINOX → gem mono | 14.8 | 13.9, 15.67 |
| Gem mono → Fluoropyr mono | 10.2 | 9.03, 12.81 |

(m)FOLFIRINOX includes both standard and modified FOLFIRINOX. (m)FOLFIRINOX, modified folinic acid, fluorouracil, irinotecan and oxaliplatin. CI, confidence interval. Fluoropyr, fluoropyrimidine. Gem, gemcitabine. Mono, monotherapy. nab-P, nab-paclitaxel. OS, overall survival.

F


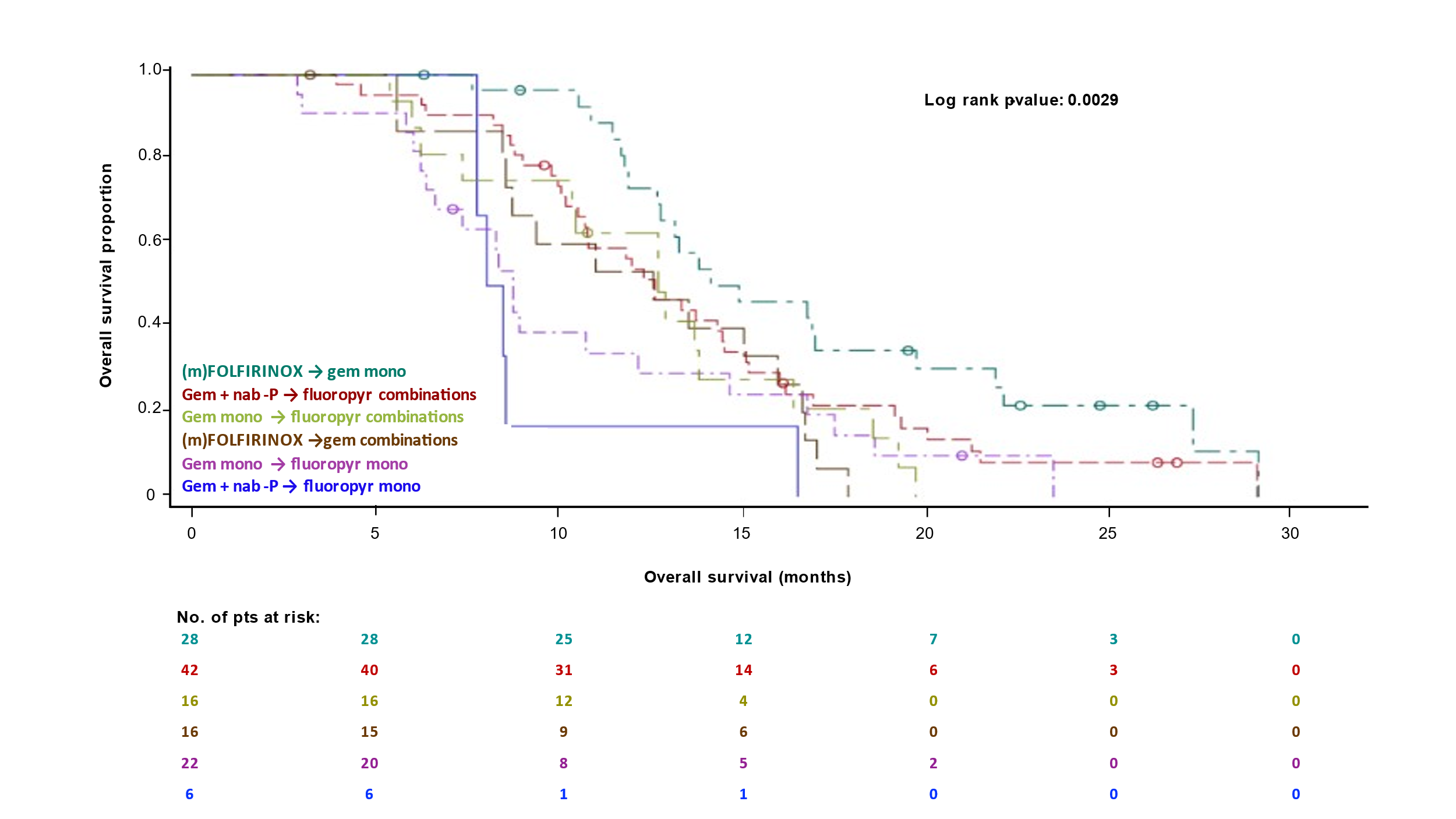


| **Treatment sequence (1L → 2L)** | **Median OS (months)** | **95% CI** |
| --- | --- | --- |
| (m)FOLFIRINOX → gem mono | 15.0 | 12.75, 19.84 |
| Gem + nab-P → Fluoropyr combinations | 12.7 | 10.58, 14.59 |
| Gem mono → Fluoropyr combinations | 12.8 | 7.43, 16.49 |
| (m)FOLFIRINOX → gem combinations | 12.6 | 8.51, 16.03 |
| Gem mono → Fluoropyr mono | 8.8 | 6.44, 12.22 |
| Gem + nab-P → Fluoropyr mono | 8.3 | 7.82, 16.59 |

(m)FOLFIRINOX includes both standard and modified FOLFIRINOX. (m)FOLFIRINOX, modified folinic acid, fluorouracil, irinotecan and oxaliplatin. CI, confidence interval. Fluoropyr, fluoropyrimidine. Gem, gemcitabine. Mono, monotherapy. nab-P, nab-paclitaxel. OS, overall survival.

**Supplementary Figure 2.** Kaplan-Meier curves for progression-free survival (A) in the 1L population with ECOG PS 0 or 1 (n=2127), (B) in the 1L population with ECOG PS ≥2 (n=1153), (C) in the 2L population with ECOG PS 0 or 1 (n=563), and (D) in the 2L population with ECOG PS ≥2 (n=584)

A


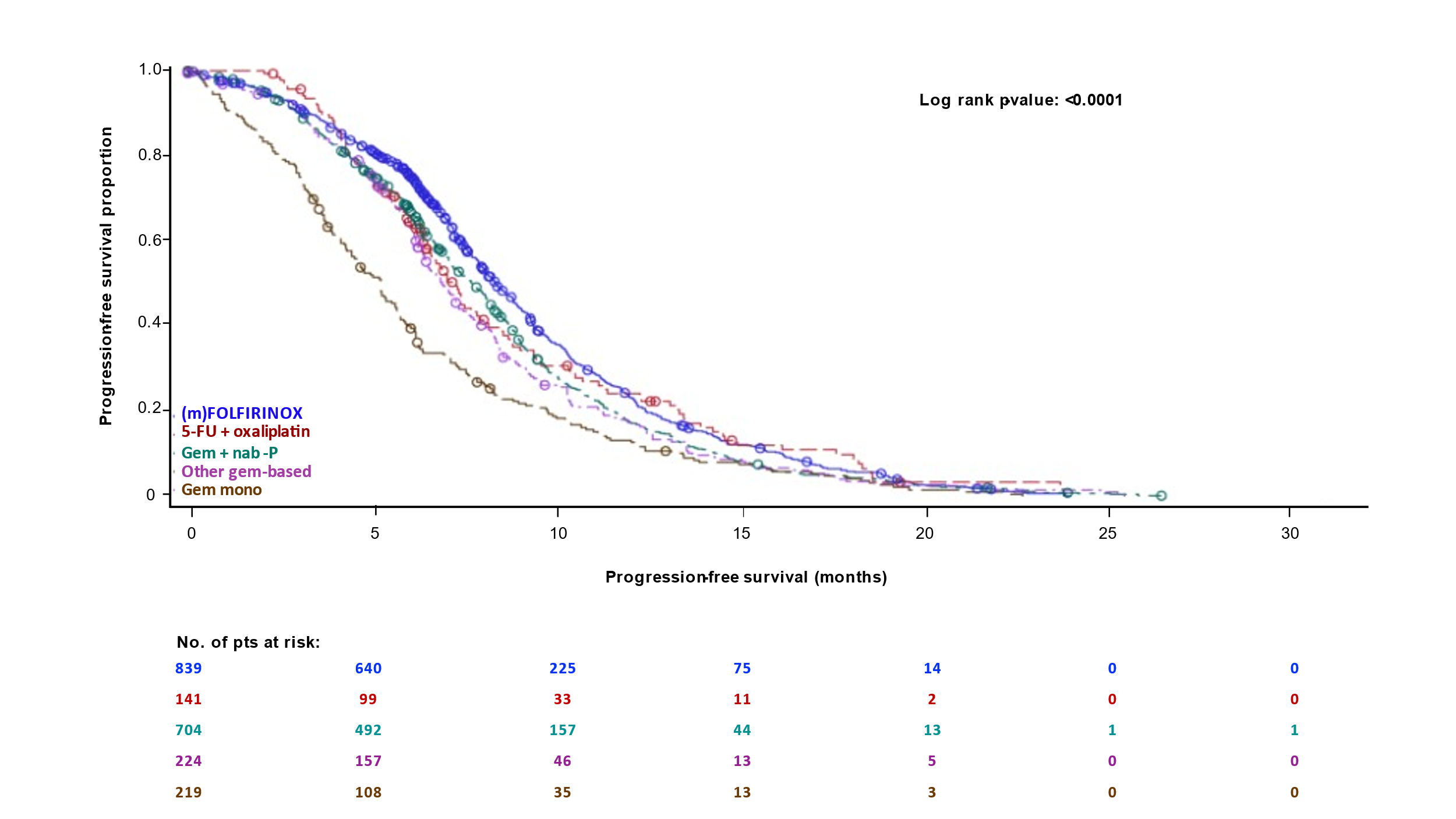


| **Treatment regimen** | **Median PFS (months)** | **95% CI** |
| --- | --- | --- |
| (m)FOLFIRINOX | 8.1 | 7.79, 8.54 |
| 5-FU + oxaliplatin | 7.0 | 6.24, 7.89 |
| Gem + nab-P | 7.5 | 7.00, 7.92 |
| Other gem-based combinations | 6.7 | 6.21, 7.33 |
| Gem mono | 5.1 | 4.34, 5.52 |

(m)FOLFIRINOX includes both standard and modified FOLFIRINOX. (m)FOLFIRINOX, modified folinic acid, fluorouracil, irinotecan and oxaliplatin. 5-FU, fluorouracil. CI, confidence interval. Gem, gemcitabine. Mono, monotherapy. nab-P, nab-paclitaxel. PFS, progression-free survival.

B


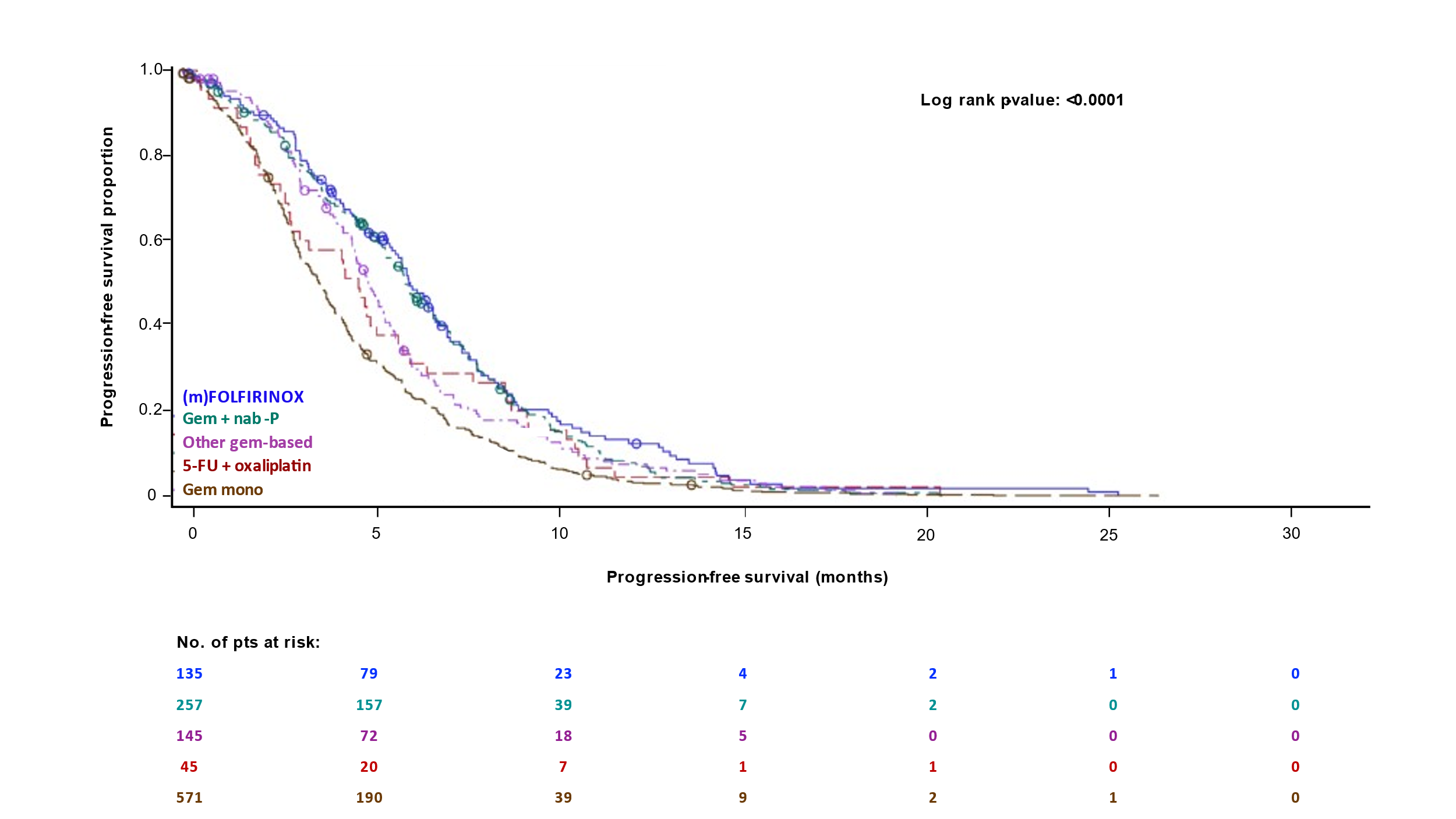


| **Treatment regimen** | **Median PFS (months)** | **95% CI** |
| --- | --- | --- |
| (m)FOLFIRINOX | 6.2 | 5.68, 6.93 |
| Gem + nab-P | 6.1 | 5.59, 6.74 |
| Other gem-based combinations | 5.1 | 4.67, 5.55 |
| 5-FU + oxaliplatin | 4.8 | 2.96, 5.91 |
| Gem mono | 3.7 | 3.45, 4.01 |

(m)FOLFIRINOX includes both standard and modified FOLFIRINOX. (m)FOLFIRINOX, modified folinic acid, fluorouracil, irinotecan and oxaliplatin. 5-FU, fluorouracil. CI, confidence interval. Gem, gemcitabine. Mono, monotherapy. nab-P, nab-paclitaxel. PFS, progression-free survival.

C


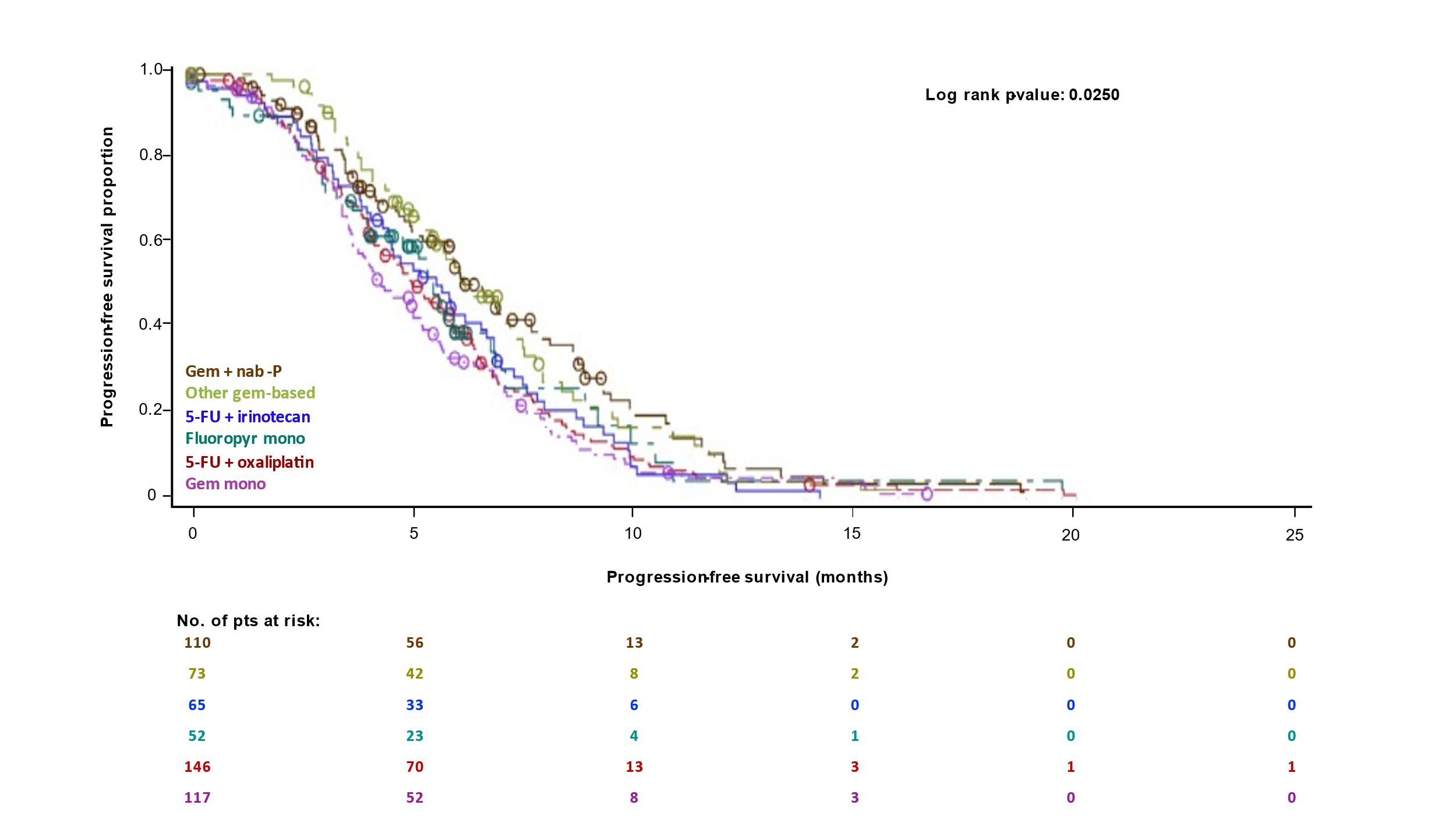


| **Treatment regimen** | **Median PFS (months)** | **95% CI** |
| --- | --- | --- |
| Gem + nab-P | 6.7 | 5.32, 7.79 |
| Other gem-based combinations | 6.3 | 5.55, 7.33 |
| 5-FU + irinotecan | 5.7 | 4.53, 6.93 |
| Fluoropyr mono | 5.6 | 4.04, 6.97 |
| 5-FU + oxaliplatin | 5.3 | 4.37, 5.98 |
| Gem mono | 4.4 | 3.78, 5.29 |

(m)FOLFIRINOX includes both standard and modified FOLFIRINOX. (m)FOLFIRINOX, modified folinic acid, fluorouracil, irinotecan and oxaliplatin. 5-FU, fluorouracil. CI, confidence interval. Fluoropyr, fluoropyrimidine. Gem, gemcitabine. Mono, monotherapy. nab-P, nab-paclitaxel. PFS, progression-free survival.

D


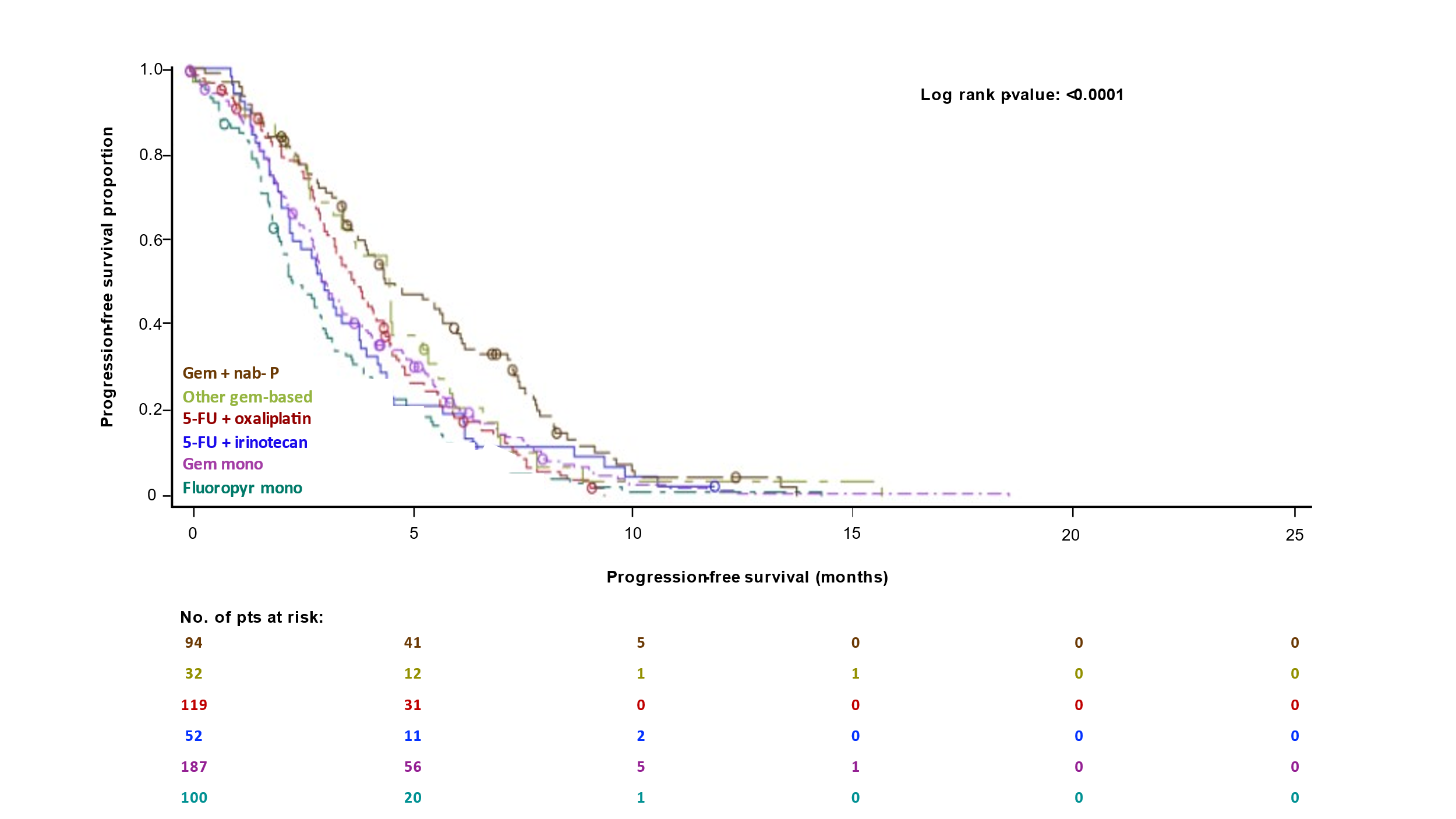


| **Treatment regimen** | **Median PFS (months)** | **95% CI** |
| --- | --- | --- |
| Gem + nab-P | 4.5 | 3.84, 6.01 |
| Other gem-based combinations | 4.6 | 2.76, 5.45 |
| 5-FU + oxaliplatin | 3.8 | 3.32, 4.27 |
| 5-FU + irinotecan | 3.1 | 2.30, 3.88 |
| Gem mono | 3.1 | 2.89, 3.52 |
| Fluoropyr mono | 2.4 | 2.07, 3.06 |

(m)FOLFIRINOX includes both standard and modified FOLFIRINOX. (m)FOLFIRINOX, modified folinic acid, fluorouracil, irinotecan and oxaliplatin. 5-FU, fluorouracil. CI, confidence interval. Fluoropyr, fluoropyrimidine. Gem, gemcitabine. Mono, monotherapy. nab-P, nab-paclitaxel. PFS, progression-free survival.

**Supplementary Figure 3.** Schoenfeld residual plots for potential variables not meeting the proportional hazard assumption from the interaction test: 1L therapy. (A) 1L (m)FOLFIRINOX; (B) 1L 5-FU + oxaliplatin; (C) 1L gemcitabine + nab-paclitaxel; (D) 1L gemcitabine monotherapy; (E) female gender; (F) ECOG PS 1.
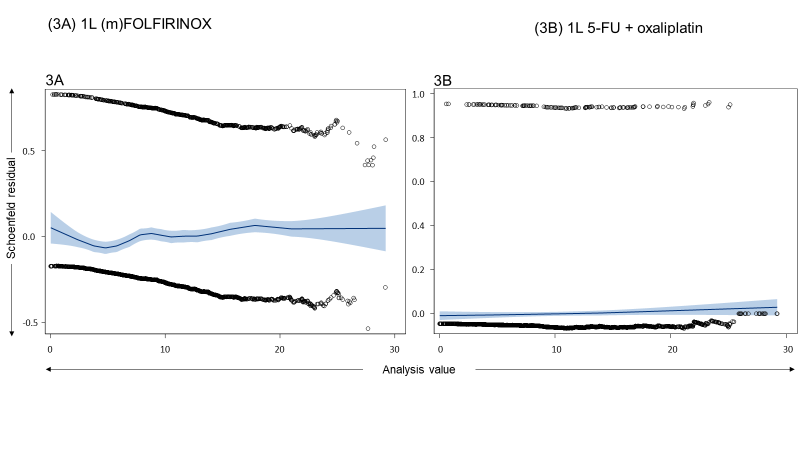

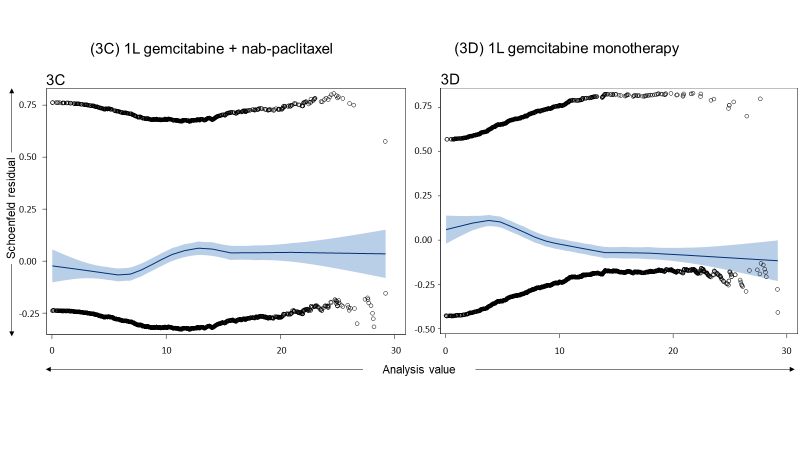

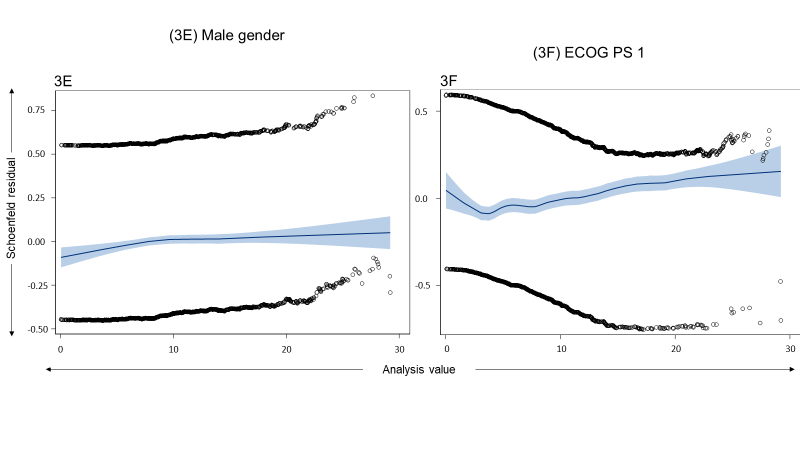
 Central dark blue line represents Loess curve; pale blue band around the dark blue line is the 95% confidence interval. Loess curves that generally remain flat confirm the Cox proportional hazards assumption; curves that do not have potential minor violations of the proportional hazards assumption.

**Supplementary Figure 4.** Schoenfeld residual plots for potential variables not meeting the proportional hazard assumption from the interaction test: 2L therapy. (A) disease grade; (B) ECOG PS 2.
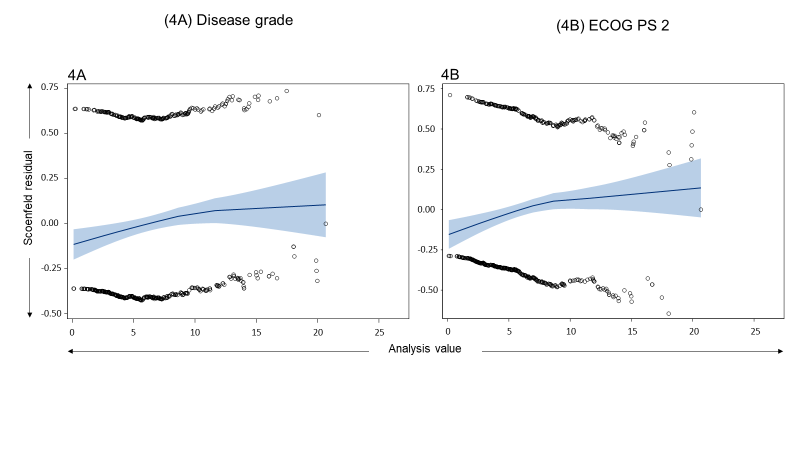
 Central dark blue line represents Loess curve; pale blue band around the dark blue line is the 95% confidence interval. Loess curves that generally remain flat confirm the Cox proportional hazards assumption; curves that do not have potential minor violations of the proportional hazards assumption.

**Supplementary Figure 5.** Schoenfeld residual plots for covariables: 1L→2L therapy. (A) 1L (m)FOLFIRINOX→2L gemcitabine monotherapy; (B) 1L (m)FOLFIRINOX→2L gemcitabine-based combinations; (C) 1L gemcitabine + nab-paclitaxel→2L fluoropyrimidine monotherapy; (D) 1L gemcitabine + nab-paclitaxel→2L fluoropyrimidine-based combinations; (E) 1L gemcitabine monotherapy→2L fluoropyrimidine monotherapy; (F) age; (G) female gender; (H) BMI <18.5 kg/m^2^; (I) BMI 18.5-25 kg/m^2^; (J) lung metastasis; (K) liver metastasis; (L) disease grade; (M) morbidity; (N) CA19-9 ≥400 U/ml; (O) ECOG PS 1; (P) tumour location.
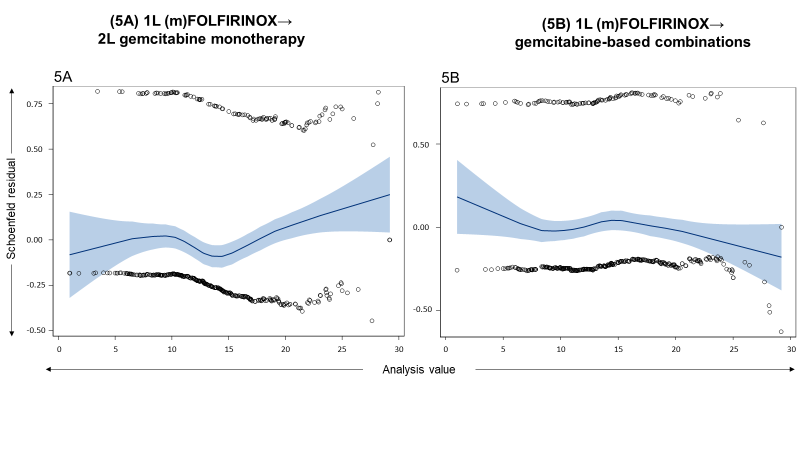

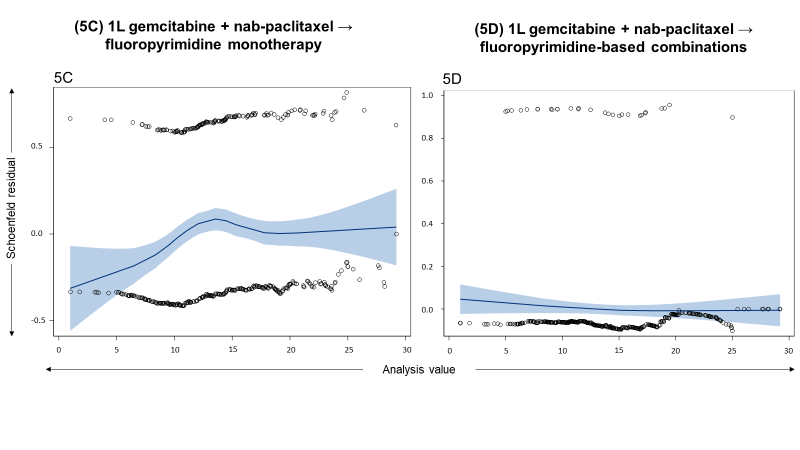

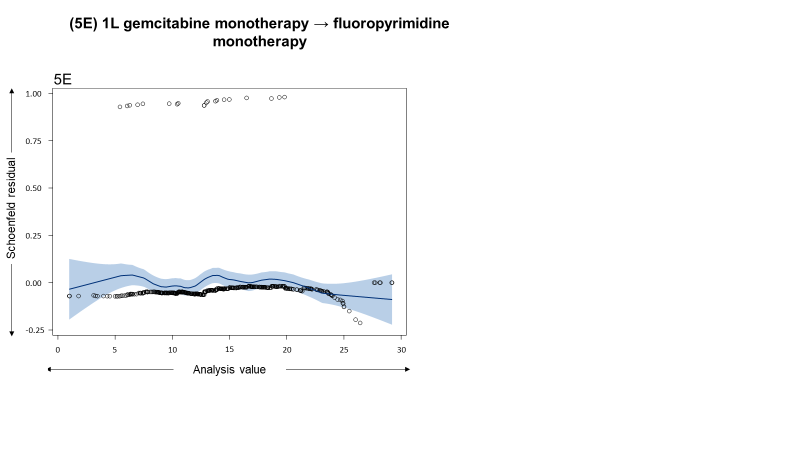

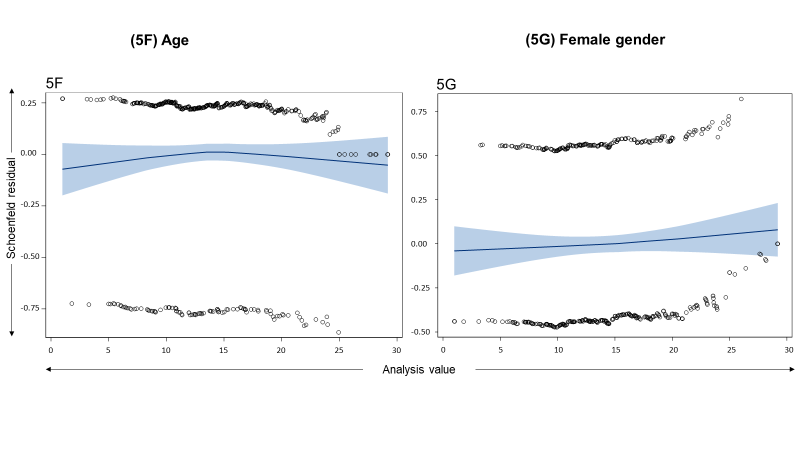

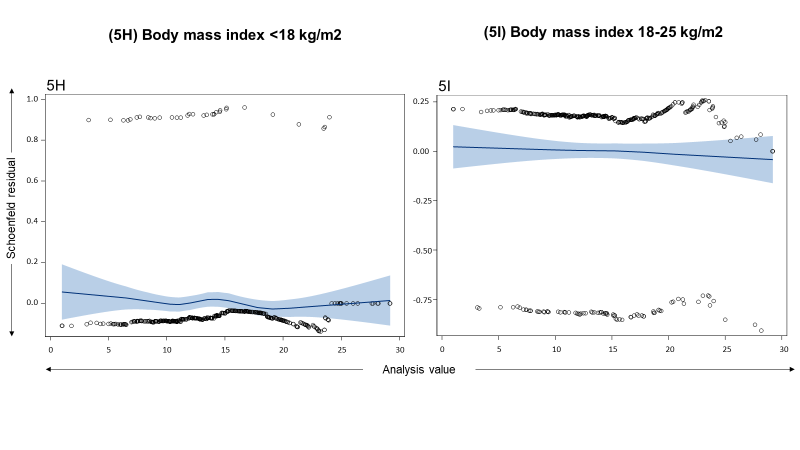

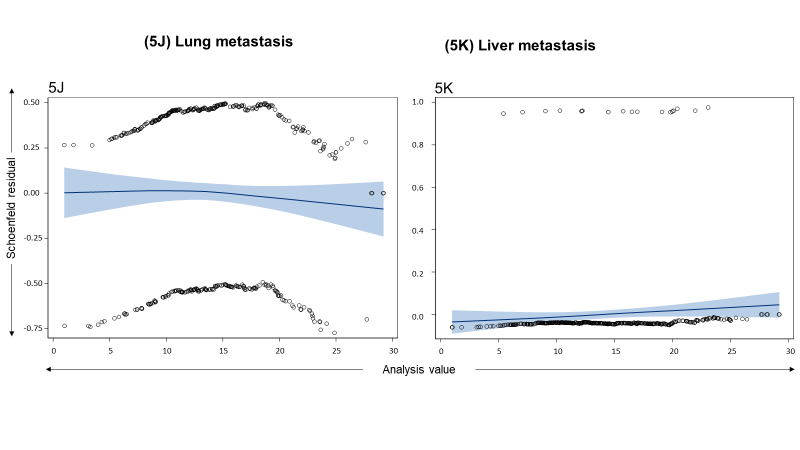

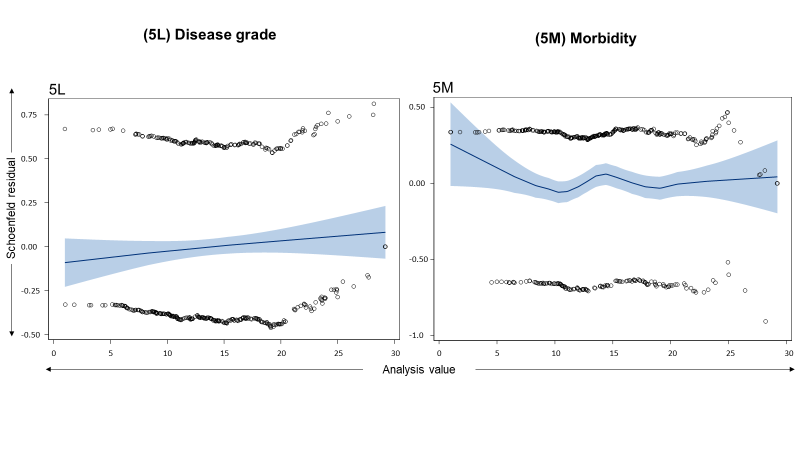

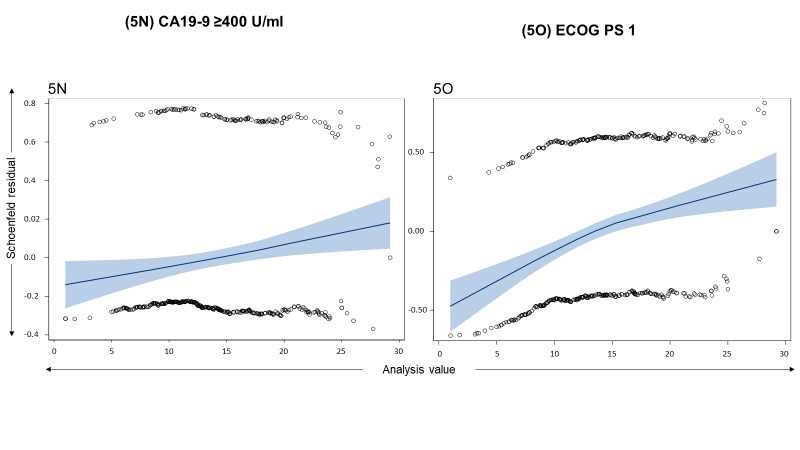


Central dark blue line represents Loess curve; pale blue band around the dark blue line is the 95% confidence interval. Loess
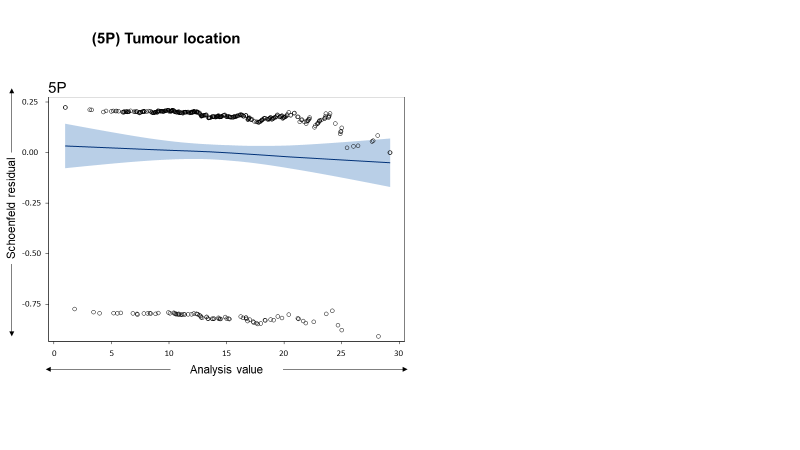
curves that generally remain flat confirm the Cox proportional hazards assumption; curves that do not have potential minor violations of the proportional hazards assumption.
